# Metabolomic profiling in heart failure as a new tool for diagnosis and phenotyping

**DOI:** 10.1038/s41598-025-95553-2

**Published:** 2025-04-07

**Authors:** Maria V. Kozhevnikova, Yuri N. Belenkov, Ksenia M. Shestakova, Anton A. Ageev, Pavel A. Markin, Anastasiia V. Kakotkina, Ekaterina O. Korobkova, Natalia E. Moskaleva, Ivan V. Kuznetsov, Natalia V. Khabarova, Alexey V. Kukharenko, Svetlana A. Appolonova

**Affiliations:** 1 Hospital Therapy No. 1 Department, Federal State Autonomous Educational Institution of Higher Education I.M. Sechenov First Moscow State Medical University of the Ministry of Health of the Russian Federation (Sechenov University), Moscow, 119435 Russia; 2 Laboratory of Pharmacokinetics and Metabolomic Analysis, Institute of Translational Medicine and Biotechnology, Federal State Autonomous Educational Institution of Higher Education I.M. Sechenov First Moscow State Medical University of the Ministry of Health of the Russian Federation (Sechenov University), Moscow, 119435 Russia; 3 I.M. Sechenov First Moscow State Medical University, 2-4 Bolshaya Pirogovskaya St., 119991 Moscow, Russia

**Keywords:** Metabolomics, Cardiovascular diseases, Heart failure

## Abstract

Classifying heart failure (HF) by stages and ejection fraction (EF) remains a debated topic in cardiology. Metabolomic profiling (MP) offers a means to identify unique pathophysiological changes across different phenotypes, presenting a promising approach for the diagnosis and prognosis of HF, as well as for the development of targeted therapies. In our study, MP was performed on 408 HF patients (54.9% male). The mean ages of patients were 62 [53;68], 67 [65;74], 68 [61;72], and 69 [65;73] years for stages A, B, C, and D, respectively. This study demonstrates high accuracy in HF stage classification, distinguishing Stage A from Stage B with an AUC ROC of 0.91 and Stage B from Stage C with an AUC ROC of 0.97, by integrating chromatography-mass spectrometry data through multiparametric machine learning models. The observed metabolic similarities between HF with mildly reduced EF and HF with reduced EF phenotypes (AUC ROC 0.96) once again highlight the fundamental differences at the cellular and molecular levels between HF with preserved EF and HF with EF < 50%. Hierarchical clustering based on MP identified four distinct HF phenotypes and 26 key metabolites, including metabolites of tryptophan catabolism, glutamine, riboflavin, norepinephrine, serine, and long- and medium-chain acylcarnitines. The average follow-up period was 542.37 [16;1271] days. A downward change in the trajectory of EF [HR 3,008, 95% CI 1,035 to 8,743, *p* = 0,043] and metabolomic cluster 3 [HR 2,880; 95% CI 1,062 to 7,810, *p* = 0,0376] were associated with increased risk of all-cause mortality. MP can refine HF phenotyping and deepen the understanding of its underlying mechanisms. Metabolomic analysis illuminates the biochemical landscape of HF, aiding in its classification and suggesting new therapeutic pathways.

**Supplementary Information:**

The online version contains supplementary material available at 10.1038/s41598-025-95553-2.

## Introduction

Heart failure (HF) is a complex and heterogeneous syndrome influenced by a wide range of etiological factors, myocardial remodeling patterns, and regulatory system activation. The disease trajectory, prognosis, and treatment options are largely determined by the specific HF phenotype, making classification a critical aspect of clinical management. To standardize classification approaches, regulatory guidelines issued in 2021 categorize HF based on stages, left ventricular ejection fraction (LVEF), and functional class^1^. LVEF-based classification divides HF into three primary groups: HF with reduced ejection fraction (HFrEF), HF with preserved ejection fraction (HFpEF), and HF with mildly reduced ejection fraction (HFmrEF). However, HFmrEF remains a “gray zone”, sharing characteristics with both HFrEF and HFpEF, making its classification and management challenging. Moreover, modern therapy can significantly improve LVEF, leading to patient transitions between phenotypes over time. Recent studies suggest that HFmrEF represents a transitional phenotype, either progressing toward full recovery (HF with improved EF, HFiEF) or deteriorating into worsening systolic dysfunction (Margonato et al., 2020). This distinction led the AHA/ACC/HFSA to recognize HFiEF as a separate phenotype^2^. Despite these refinements, controversies remain regarding whether HFmrEF should be treated as a distinct entity or categorized within HFrEF or HFpEF.

Although LVEF-based classification is fundamental for guiding treatment decisions, stage-based classification remains clinically relevant, particularly for identifying patients at risk of HF (pre-HF, Stages A and B). Early detection of these patients is crucial for implementing preventive strategies and ensuring close monitoring to delay disease progression.

Despite their clinical utility, current classification schemes fail to fully capture the pathophysiological complexity of HF. There is growing recognition that a molecular approach—focusing on underlying biochemical and metabolic mechanisms—may enhance patient stratification and therapeutic decision-making. Omics technologies, particularly metabolomics, offer a unique opportunity to assess systemic metabolic dysregulation and provide insights into the biochemical pathways driving HF progression.

Metabolomic profiling, when integrated with existing HF classifications, may reveal novel metabolic markers that serve as both diagnostic and prognostic tools, while also refining current classification models.

This study aims to analyze metabolic pathway alterations across different HF classifications, providing a deeper understanding of HF heterogeneity and identifying potential diagnostic markers and therapeutic targets.

## Methods

### Study population

We included 408 patients with different stages of HF according to the American Heart Association/American Heart Failure Association classification^2^. Stage A was defined by absence of HF symptoms, structural heart disease, or cardiac biomarkers increase. Stage B was detected by absence of symptoms or signs of HF but presence of structural changes: LAVI ≥ 29 mL/m^2^, LVMI > 115/95 g/m^2^, relative wall thickness (RWT) > 0.42, LV wall thickness ≥ 12 mm, average E/e′ ≥15 for increased filling pressures, septal e’ <7 cm/s, lateral e’ <10 cm/s, TR velocity > 2.8 m/sec, pulmonary artery systolic pressure (PASP) > 35 mmHg or brain natriuretic peptide (BNP) ≥ 35 pg/mL, N-terminal pro-B-type natriuretic peptide (NT-proBNP) ≥ 125 pg/mL. Stage C characterized by structural heart disease with current or previous symptoms of HF (presence of signs of lung congestion or peripheral oedema, hepatomegaly, jugular venous distention). Stage D was defined by marked HF symptoms that interfere with daily life and with recurrent hospitalizations despite attempts to optimize guideline-directed medical therapy^2^. Patients with stages C and D were classified by EF (*n* = 218). HFrEF was defined by a decrease of LVEF < 40%. HF with mildly reduced EF (HFmrEF) was determined in LVEF 40–49%. HF with preserved EF (HFpEF) was verified in LVEF ≥ 50% and NT-proBNP > 125 pg/mL or at least one of the additional items: enlargement of the left atrium (LA) (LA volume index (LAVI) > 34 ml/m^2^), hypertrophy of the LV (LV mass index (LVMI) > 115 in men and > 95 g/m^2^in women), early diastolic velocity of movement of the fibrous ring of the mitral valve (e’) at the level of the interventricular septum (< 7 cm/s) and the posterior wall (< 10 cm/s), average E/e’ (≥ 15), maximum speed of tricuspid regurgitation (TR) velocity (> 2.8 m/s)^3^. Hypertension and coronary artery disease (CAD) was defined according to the guidelines^4,5^.

Exclusion criterion were: acute forms of CAD, hypertrophic cardiomyopathy, restrictive cardiomyopathy; valvular heart disease; myocarditis, pericarditis; stroke within the previous 6 months; acute renal failure, chronic kidney disease stage 5; chronic pulmonary heart disease; hepatic cellular failure; bronchial asthma, chronic obstructive pulmonary disease; gastric ulcer in the acute phase; chronic pancreatitis in the acute phase; cancer; type 1 diabetes mellitus; thrombocytopenia of any origin, hemorrhagic syndrome; chronic viral and bacterial infections; autoimmune diseases; mental illness.

The study was conducted with the Declaration of Helsinki of the World Medical Association principles, adopted at the 18th General Assembly of the WMA, was approved by the Local Ethics Committee of Sechenov University protocol №34 − 20 (09.12.2020) and was carried out by decision of the Academic Council of Sechenov University. All study participants gave written informed consent.

## Metabolomic analysis

Quantitative analysis of metabolites was performed by target chromato-mass-spectrometric method according to with some modifications^6^.

10 µL of the blood plasma sample (or calibration standards and quality control (QC) samples) and 40 µL of ISTD mix in methanol were pipetted in Eppendorf tubes. After drying the samples in vacuum concentrator 50 µL of PITC derivatization solution (5% phenyl isothiocyanate in the mixture of acetonitrile, water and pyridine, 1:1:1) was added and kept at room temperature for 20 min. After another 1-h drying in vacuum concentrator 100 µL of methanol with 5mM ammonium acetate and 100 µL of water were added to each sample. After 30 min extraction samples were centrifuged at 13,000 rpm for 10 min and supernatant was analyzed by liquid chromatography mass spectrometry (LC-MS/MS).

An Agilent 1200 series HPLC system and Agilent 6460 triple-quadruple mass-spectrometer (Palo Alto, CA, U.S.A.) were used for LC-MS/MS analysis. Acquity UPLC BEH C18 2,1 × 50 mm; 1,7 mcm (Waters Corp., U.S.A) column with Acquity UPLC BEH C18 (2,1 × 5 mm; 1,7 mcm) precolumn was used for chromatographic separation. The UPLC parameters were as follows: solvent A 0.1% (v/v) formic acid in water, and solvent B 0.1% (v/v) formic acid in acetonitrile. The column temperature was maintained at 40 °C. The gradient program was as follows: 0.5 min − 1% B, 1 min – 30% B, 3 min − 75% B, 3,5 min − 95% B, 4.5 min − 95% B, 4,6 min − 1% B, 6 min − 1% B. Flow rate was 500 µL/min, and the sample injection volume was 10 µL.

A mass spectrometric detector with a triple quadrupole in the scheduled MRM mode was used for analysis (Table S1). For Agilent Jet stream ion source the capillary voltage was 3500 V and nebulizer pressure was 30 psi. The Gas Temp, Gas Flow, Sheath Gas Heater and Sheath Gas Flow were 300 °C, 11 l/min, 300 °C and 11 l/min, respectively. The fragmentor voltage, collision energy, MRM precursor ion (Q1), and fragment ion (Q3) were optimized and set individually for each analyte and isotope-labeled ISTD.

Quantitative analyses were done in MassHunter ver. B.08.00 software (Agilent, Palo Alto, CA, U.S.A.) (https://www.agilent.com/en/product/software-informatics/mass-spectrometry-software/data-analysis/quantitative-analysis) based on the peak area ratios of the targeted analyte compared to its isotope-labeled ISTD. Calibration regression was built for each analyte within its plasma concentration range. For acylcarnitine analysis quantification was done by semiquantitative method based on the peak area ratios of the target analyte and its isotope-labeled standards only without calibration curves.

Methods were validated for selectivity, linearity, precision, accuracy, recovery, matrix effect and stability according to US FDA and EMA guidelines for bioanalytical method validation (EMA, 2019; USFDA, 2018). Detection limit and linear range are selected for each compound according to physiological plasma concentrations.

### Clinical follow-up

Patients with symptomatic HF (*n* = 218) had a follow-up period. The primary outcomes were all cause death. The average follow-up period was 542.37 [16;1271] days. When multiple events occurred, patients were censored at the time of the first event. Follow-up events were adjudicated by an independent trained investigator.

### Statistical analysis

Quantitative indicators were assessed for compliance with normal distribution using the Shapiro-Wilk test (if the number of subjects was less than 50). Comparison of two groups according to a quantitative indicator with a normal distribution, provided that the variances were equal, was performed using the Student t-test, and when the variances were unequal, there was applied the Welch t-test. Comparison of two groups for quantitative indicators whose distribution differed from normal was performed using the Mann-Whitney U test. Comparison of several groups on a quantitative indicator with a normal distribution was performed using one-way analysis of variance, post-hoc comparisons were carried out using Fisher’s test (assuming equal variances) and Welch’s test (if unequal variances). To compare quantitative indicators of several groups, the distribution of which differed from normal, the nonparametric Kruskal-Wallis test was used. If statistically significant differences were identified, further post hoc pairwise comparison of groups was carried out using Dunn’s test with Holm’s correction. Pearson chi-square was used to compare categorical variables across multiple independent groups. Differences were considered significant at *p* < 0.05. The direction and strength of the correlation between two quantitative indicators were assessed using the Pearson correlation coefficient (if the compared indicators were normally distributed) or the Spearman correlation coefficient (if the compared indicators were not normally distributed). The connection more than 0.3 were taking into account. Patients’ survival function was assessed using the Kaplan-Meier method. The analysis of patient survival was carried out using the Cox regression method, which involves predicting the risk of an event for the object in question and assessing the influence of predetermined independent variables (predictors) on this risk. Risk is viewed as a time-dependent function. Missing data were not included in the models. Data were analyzed with SPSS version 25.0 (IBM Corp., Armonk, NY, USA) and R 3.6.2 (R Foundation for Statistical Computing, Vienna, Austria).

Raw LC-MS/MS data preprocessing included normalization and replacement of outliers and missing values via pandas (v.2.2.3), numpy (v.2.1.2), scipy (v.1.14.1) packages in python (v.3.11). QC samples were analyzed each ten samples to control stability of the instrument and to normalize the variations in analyzed batch.

Further principal component analysis was applied to explore trends in the results of metabolomic profiling in the desired groups of patients. Additionally, to assess the diagnostic accuracy of different classifications of HF stages ML models based on the random forest algorithms were built. For this reason, the desired datasets were randomly divided into discovery (70%) and test datasets (30%). Hyperparameters for the random forest models was tuned using cross-validated (k = 30) GridSearchCV function.

The random forest algorithm is a nonparametric ensemble method is based on a combination of several decision trees, called “random trees”. Each tree is built based on a random subsample of the training data and a random subset of features, that implements “randomness” into the learning of each tree. It serves for the reduction of the correlation between trees and increases the diversity of models.

The assessment of classification quality was performed using common quality metrics, including confusion matrix, area under the receiver operating characteristics curve (AUC ROC), accuracy, f1-score and recall. The principal components analysis (PCA), random forest models were performed using the scikit-learn package (v.1.5.2) in Python (v.3.11).

Hierarchal cluster analysis (HCA) is a clustering method that explores the organization of samples into groups therefore providing a hierarchy usually represented as a dendrogram. Here, the HCA was performed using the Ward minimum variance method and preliminary reduction of the data dimensionality to nine principal components via Simca-P + software (version 14.1, Umetrics) (URL: https://www.sartorius.com/en/products/process-analytical-technology/data-analytics-software/mvda-software/simca).

## Results

### Study population

Demographic and clinical characteristics of all subjects are shown in Table 1. Stage A was presented predominantly by hypertensive patients and coronary artery disease (CAD) patients (130 patients, 31.9%). The stage B was represented predominantly by patients with CAD and to a lesser extent by patients with hypertension (60 patients, 14.7%). HFrEF had 81 patients (37.0%), HFpEF was detected in 87 patients (40.0%), HFmrEF was detected in 50 patients (23%).

**Table 1:** Population characteristics.

Variable	Stage A (*n* = 130)	Stage B (*n* = 60)	Stage C (*n* = 118)	Stage D (*n* = 100)	*P*-value
Demographics					
Age, years	62 [53–68]	67 [65–74] *	68 [61–72]*	69 [65–73]*	**< 0.000001**
Male sex, n (%)	60 (46.2)	32 (53.3)	70 (59.3)	62 (62.0)	0.07
BMI, kg/m^2^	30 [26–32]	32 [28–25]	32 [27–36]*	31 [28–35]	**< 0.009**
Smoking, n (%)	30 (25.2)	12 (20.0)	25 (21.4)	12 (12.0)	0.10
**Clinical evaluation**					
Obesity	58 (44.6)	40 (66.7) *	67 (56.8)	53 (53.0)	**0.03**
Arterial hypertension	99 (76.2)	50 (83.3)	117 (99.1)	100 (100)	**< 0.0001**
Dyslipidaemia^***a***^	99 (76.1)	49 (83.1) *	100 (84.7) *	71 (71.0) ***	**< 0.000001**
Impaired glucose tolerance	14 (11.9)	11 (18.3)	20 (16.9)	21 (21.0)	
Diabetes mellitus	25 (21.2)	17 (28.3)	43 (36.4) *	49 (49.0) ***	**< 0.00002**
Stroke/TIA	1 (2.1)	8 (18.6)	20 (16.9)	16 (16.0)	0.06
CAD	34 (26.2)	48 (80.0)	50 (83.3)	100 (100.0)	**< 0.0001**
PCI	12 (9.2)	22 (36.7) *	19 (16.1) * *	27 (27.0) *	**< 0.00002**
CABG	2 (4.4)	3 (6.4)	8 (10.3)	5 (5.0)	0.49
Previous MI	10 (10.4)	26 (46.4) *	48 (61.5) *	77 (77.0) ***	**< 0.000001**
HFpEF	0	0	69 (58.5)	18 (18.0)	**< 0.000001**
HFmrEF	0	0	21 (17.8)	29 (29.0)	
HFrEF	0	0	28 (23.7)	53 (53.0)	
**Blood tests**					
Haemoglobin, g/L	141.0 [132.0–153.0]	144.0 [133.0–154.0]	138 [129–148.0]	132 [119.75–144.0] *,**	**0.000006**
WBC, *10^9^	6.2 [5.4–7.53]	7.2 [5.97–8.81] *	7.8 [6.4–9.17]*	8.0 [6.8–9.65]*^,^**	**< 0.00001**
K^+^, mMol/L	4.6 [4.38–4.90]	4.5 [4.30–4.90]	4.5 [4.30–4.97]	4.4 [4.0–4.7]*,***	**0.00058**
Total cholesterol, mmol/L	5.27 ± 1.28	4.57 ± 1.36*	4.35 ± 1.37*	3.64 ± 1.08*,** ***	**< 0.000001**
LDL, mg/dL	3.25 [2.44–3.75]	2.48 [1.88–3.27]*	2.94 [1.9–3.59]	2.13 [1.52–2.93]*,***	**< 0.0001**
HDL, mg/dL	1.42 [1.18–1.67]	1.17 [1.01–1.55]	1.04 [0.86–1.30]*	0.95 [0.72–1.23]*	**< 0.000001**
Triglycerides, mg/dL	1.3 [1.02–1.91]	1.23 [1.00–1.83]	1.36 [0.98–1.95]*	1.09 [0.82–1.40]*	**< 0.0053**
Creatinine, mkmol/L	91.95 [81.42–101.6]	97.10 [84.48–110.72]	99.5 [88.25–114.00]	111.0 [93.75–127.25] *^,^**,***	**< 0.000001**
eGFR, mL/min/1.73 m^2^	69.37 ± 15.09	62.42 ± 19.28*	60.76 ± 15.71	54.02 ± 15.82 *,**,***	**< 0.000001**
Fasting blood glucose, mmol/L	5.6 [5.2–6.3]	5.8 [5.1–6.52]*	6.0 [5.3–7.2]	6.0 [5.0–8.2]	0.165
Uric acid, mkmol/L	328.5 [274.8–385.2]	353.8 [311.2–406.9]	410.0 [311.2–482.0]*	456.0 [361.0–529.0]*^,^**,***	**< 0.000001**
CRP, mg/L	1.57 [0.66–4.00]	1.6 [0.70–3.00]	4.00 [1.50–7.10]*	9.13 [4.49–22.54]*^,^**, ***	**< 0.000001**
Ferrum, mkmol/L	17.55 [15.30–22.50]	14.10 [11.70–22.20]	14.00 [9.7–20.95]*	6.50 [4.00–14.70]*,**,***	**0.000014**
NT-proBNP, pg/mL	51 [48–56]	101 [46–143]	1300 [376–3953]*,**	3733 [1341–7250]*^,^**,***	**< 0.000001**
**Therapy**					
Beta-blocker	63 (52.5)	44 (73.3)*	101 (85.6)*	87 (87.0)*	**< 0.000001**
ACEi	67 (56.3)	34 (56.7)	50 (42.4)	30 (30.0)	**0.00036**
ARB	31 (25.8)	14 (23.3)	37 (31.4)	18 (18.0)	0.153
ARNI	0 (0.0)	0 (0.0)	27 (34.6)	49 (49.0)	**< 0.0001**
MRA	5 (5.0)	15 (25.0)	77 (65.3)	83 (83.0)	**< 0.0001**
SGLT2i	1 (2.1)	7 (16.3)	18 (15.3)	19 (19.0)	0.051
Digoxin	0	0	16 (13.6)	31 (31.0)	**0.01**
CCB	32 (26.7)	20 (33.3)	38 (32.2)	12 (12.0) *,**,***	**0.0025**
Oral anticoagulant	24 (20.0)	26 (43.3)*	75 (63.6)*	78 (78.0) *,**,***	**< 0.0001**
Statins	55 (45.8)	17 (28.3)	45 (38.1)	52 (52.0)**	**0.0178**
Furosemide	0	0	78 (66.1)	85 (85.0)	**< 0.0001**
Amiodaron	11 (9.2)	8 (13.3)	14 (11.9)	2 (2.0)**,***	**0.033**

Values are mean ± standard deviation, *n* (%), or median [25th quartile–75th quartile].

ACEi, angiotensin-converting enzyme inhibitor; ARB, angiotensin receptor blocker; ARNI, angiotensin receptor–neprilysin inhibitor; BMI, body mass index; CAD, coronary artery disease; CABG – coronary artery bypass grafting; CCB, calcium channel blocker; eGFR, estimated glomerular filtration rate; HDL, high-density lipoprotein; HFmrEF - heart failure with mildly reduced ejection fraction; HFpEF, heart failure with preserved ejection fraction; HFrEF, heart failure with reduced ejection fraction; CRP, C-reactive protein; LDL, low-density lipoprotein; MI, myocardial infarction; MRA, mineralocorticoid receptor antagonist; NT-proBNP, N-terminal pro-B-type natriuretic peptide; PCI, percutaneous coronary intervention; SGLT2i, sodium/glucose cotransporter-2 inhibitors; TIA, transient ischemic attack; WBC, white blood counts.

^*^ *P* < 0.01 vs. Stage A.

^**^ *P* < 0.01 vs. Stage B.

^***^ *P* < 0.01 vs. Stage C.

### Metabolomic analysis design and data preparation

**Fig. 1:**
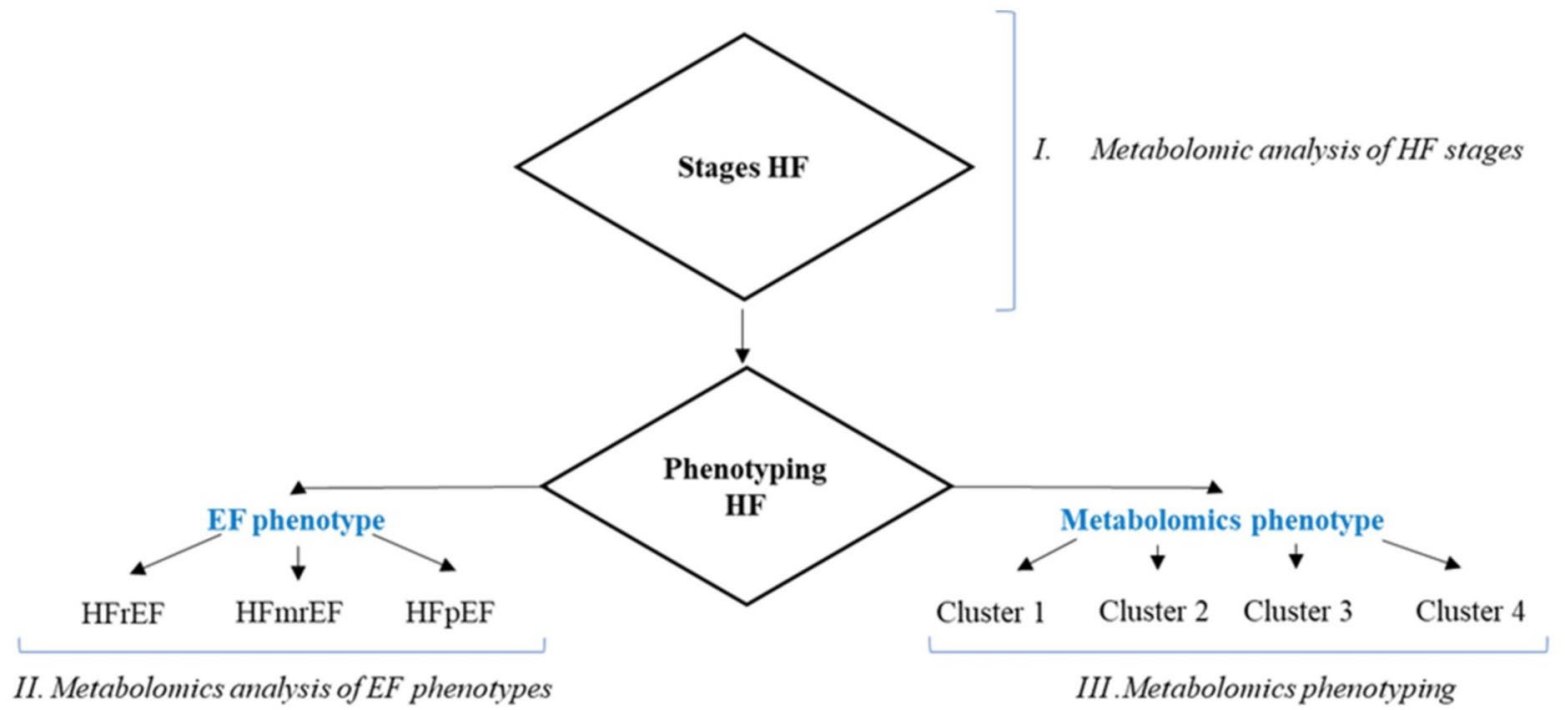
Design of the metabolomic analysis. EF - ejection fraction; HF - heart failure; HFmrEF - heart failure with mildly reduced ejection fraction; HFpEF - heart failure with preserved ejection fraction; HFrEF - heart failure with reduced ejection fraction.

To implement the objectives of the study, a metabolomic analysis design was constructed, according to which the creation of groups for comparison was determined by a specific task at each stage. At the first stage, the comparing analysis of HF stages was performed. The second stage of the analysis included only patients of the HF C and D groups, who were divided into 3 groups according to the LVEF phenotype. And, finally, at stage 3, patients with HF stage C and HF stage D were clustered according to the metabolomic profile (Fig. 1).

### Metabolomic profile in patients with different stages of HF

**Fig. 2:**
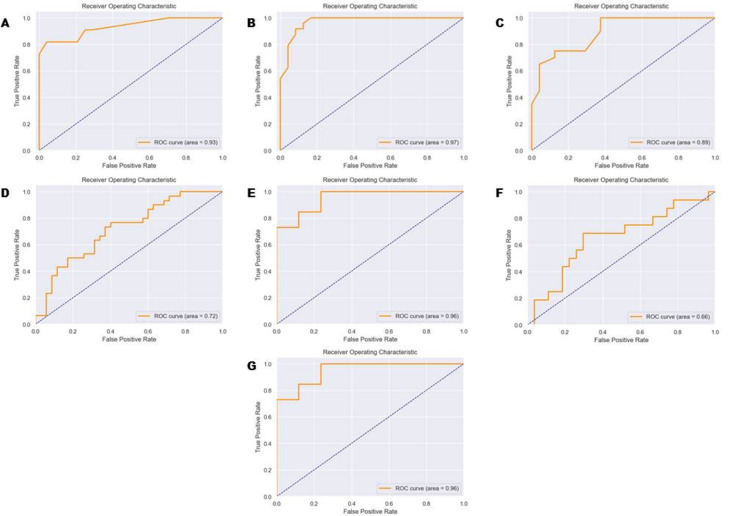
ROC curve of HF in the studied subgroups. **(A)** Classification model – Stage A vs. Stage B groups. Confusion matrix: Accuracy 0.88, Recall 0.88, AUC ROC 0.93, F1 0.88. **(B)** Classification model – Stage B vs. Stage C groups. Accuracy 0.89, Recall 0.89, AUC ROC 0.97, F1 0.89. **(C)** Classification model – Stage C vs. Stage D groups. Confusion matrix: Accuracy 0.79, Recall 0.79, AUC ROC 0.89, F1 0.79. **(D)** Classification model – HFpEF vs. HFmrEF vs. HFrEF. Confusion matrix: Accuracy 0.66, Recall 0.66, AUC ROC 0.72, F1 0.64. **(E)** Classification model - HFpEF vs. HF with EF < 50%. Confusion matrix: Accuracy 0.88, Recall 0.88, AUC ROC 0.96, F1 0.88. **(F)** Classification model - HFrEF vs. HF with EF > 40%. Confusion matrix: Accuracy 0.70, Recall 0.70, AUC ROC 0.66, F1 0.68. **(G)** Classification model – Cluster 1 vs. Cluster 2 vs. Cluster 3 vs. Cluster 4. Confusion matrix: Accuracy 0.95, Recall 0.95, AUC ROC 0.96, F1 0.95.

The conducted principal component analysis showed weak differences in the four desired groups of patients (Figure S1). Further we have learned the machine learning (ML) classification model to assess the possibility of separation HF patients in according to the provided methodology. The utilized ML algorithm represented random forest classifier with the hyperparameters and quality control metrics presented in the Table S2 and S3, respectively. Through the following deeper investigation of the data, we also assessed the diagnostic power between patients from Stage A vs. Stage B groups (Fig. 2A), Stage B vs. Stage C groups (Fig. 2B), and Stage C vs. Stage D groups (Fig. 2C). The calculated AUC ROC metrics showed weak diagnostic power for separating patients between stage C and stage D. Data from the most significant metabolites in all models are reported in Table S4-S6. This result demonstrated that classification models of ML can accurately predict the presence of stage B od stage C.

### Metabolomic profile in patients with different EF phenotypes

Patients from stage C and stage D groups were separated into three phenotypic classes in accordance to the EF and labeled as HFpEF, HFmrEF and HFrEF.^3^ The assessment of the possible application of such classification was performed using the ML modelling as is represented above. Diagnostic quality of the multiclass model is presented in Fig. 2D. The quality metrics showed weak diagnostic accuracy of the model thus we made a hypothesis that metabolomic profile in HFmrEF can be similar to HFpEF or HFrEF types. So, two classification models were found: HFpEF vs. HF with EF < 50% and HFrEF vs. HF with EF > 40%. The first model (HFpEF vs. HF with EF < 50%) had good precision (Fig. 2E), while the model HFrEF vs. HF with EF > 40% had poor accuracy (Fig. 2F). These results suggested support the model HFpEF vs. HF with EF < 50%. The key metabolites forming the classification model of HFpEF vs. HF with EF < 50% presented in the Table S7.

### Clustering by metabolomic profile

**Fig. 3:**
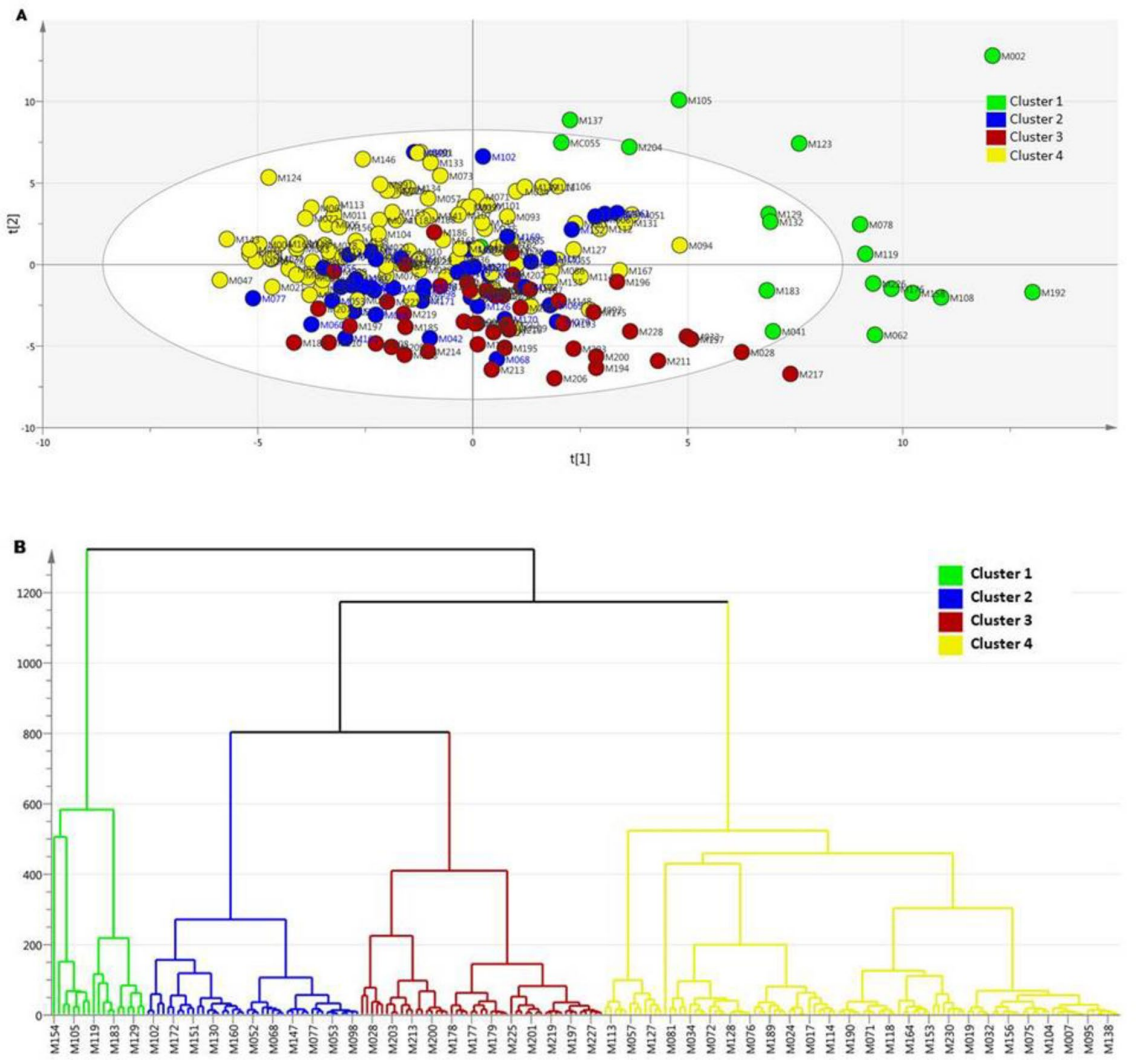
Clustering of patients with heart failure by metabolomic profile. **(A)** PCA by metabolomic profile. **(B)** Hierarchical clustering by metabolomic profile. PCA - principal components analysis.

Alternatively, we applied the cluster analysis method to identify new classification based on the results of the plasma metabolomic profiling. In this case, we performed hierarchical clustering by metabolomic profile based on the first main component that divided all patients with symptomatic HF (stage C and D) into four clusters (Fig. 3A and B). The new classification model had AUC ROC 0.96 with metrics: max_depth = 30, max_features = 20, min_samples_leaf = 1, n_estimators = 20, random_state = 42. (Fig. 2G). Further, the 26 most significant metabolites for this classification were identified, belonging mainly to the class of acylcarnitine’s, metabolites of the tryptophan-kynurenine and tryptophan-serotonin pathways, and several amino acids (Table S8).

**Fig. 4:**
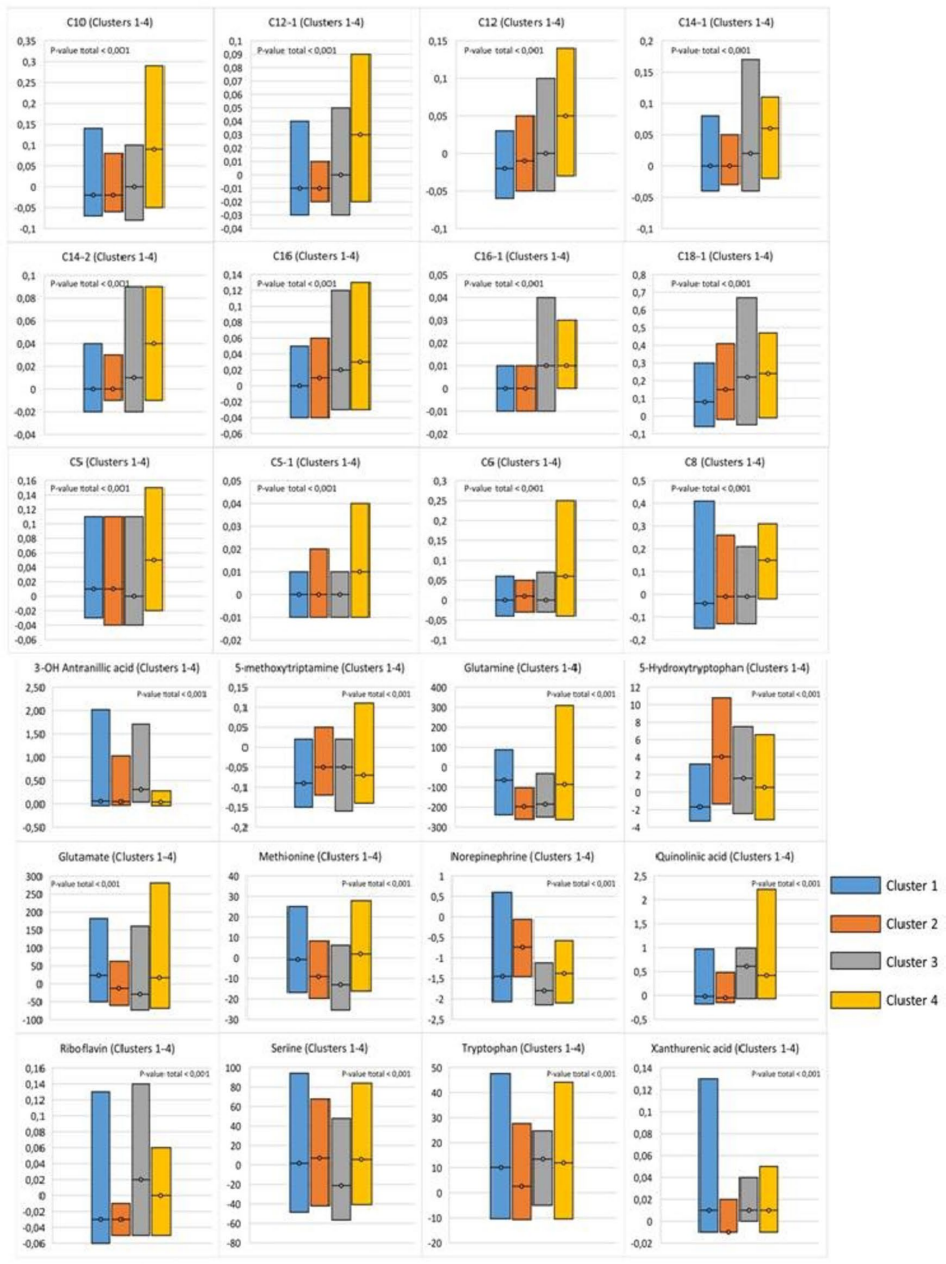
Comparative analysis of significant metabolites in the classification model of clustering.

Thus, it was possible to divide all patients with HF into 4 clusters using biostatistical processing of the metabolomic profile. In this classification, 26 metabolites demonstrated the greatest significance. Further, to assess the nature of metabolic disorders characteristic of each cluster, a comparative analysis of 4 clusters for significant metabolites was carried out. Metabolites of the kynurenin pathway of tryptophan catabolism (3-OH-anthranilic acid, quinolinic acid, and xanthurenic acid) were significantly increased in cluster 3 compared to other clusters. At the same time, serotonin pathway metabolites (5-hydrocytryptophan, 5-methoxytryptamine) were significantly reduced in cluster 1 compared to the other groups. Glutamine was significantly reduced in the second group and the third clusters compared to clusters 1 and 4. An increase in riboflavin was characteristic of cluster 3. Norepinephrine was statistically significantly elevated in cluster 2 compared to all groups studied, while cluster 3 had the lowest values. Cluster 3 was characterized by a decrease in serine compared to other subgroups. Finally, long- and medium-chain acylcarnitine, as well as the isovalerylcarnitine, tiglylcarnitine, and glutarylcarnitine metabolites, were statistically significantly higher in cluster 4 compared to the other clusters (Fig. 4, Table S9).

### Survival of patients with chronic heart failure

During a median follow-up of 542.37 [16; 1271] days, 57 (26.14%) patients had outcomes event (all causes death), the annual mortality rate was 14% (*n* = 31). For the Kaplan-Meier assay, patients who discontinued follow-up within 1 month of hospital discharge were excluded from the analysis (*n* = 24). The 25th percent was 481.64 days. The analysis showed that the overall survival rate at 2 years was 74.8%. At the same time, half of the deaths occurred in the first 2 months of follow-up (*n* = 29). The three-year risk of death in patients with HF was 41.9%.

The study design involved only telephone contact with the patient at the end of follow-up to assess outcomes development. However, information from the medical records of patients from the HF group who regularly sought medical care at the clinical sites of the study during the follow-up period was analyzed (*n* = 98). It was found that 49 (50%) patients did not show significant deviations in LVEF levels during follow-up (less than 5%), 16 (16.3%) patients had a decrease in EF, and 33 (33.7%) patients showed improvement or recovery of LVEF during treatment. In the analysis of the therapy taken, it was found that 8 patients (8.2%) patients regularly use the GDMT, 31 (31.6%) of the patient used only two classes of recommended therapy, the remaining 59 (60.2%) patients used 3 of the four recommended classes of drugs. GDMT treatment group had a better survival rate of 87.5% compared to patients taking 3 classes of drugs (54.9%) and patients taking 2 classes of drugs (52.0%). During follow-up, myocardial infarction developed in 8 (8.2%) patients, 7 of whom underwent percutaneous coronary intervention and stenting of the infarction-binding artery. Two patients (2%) underwent coronary artery bypass graft as planned. There was no significant association of EF changes with therapy, MI or revascularization.

**Fig. 5:**
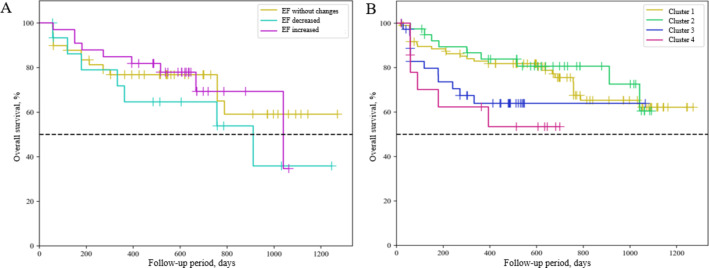
Overall survival analysis of HF in the studied subgroups. Kaplan-Meier curves of HF by EF persistent, EF decreased and EF improved (**A**) and by metabolomic cluster (**B**) subgroups. EF – ejection fraction, HF - heart failure.

To assess the course of HF in the studied subgroups (EF persistent, EF decreased, EF improved), the Kaplan-Meier analysis was carried out (Fig. 5A). The analysis showed that within 1000 days, patients with decreased LVEF had the worst prognosis, regardless of the baseline level of EF. However, survival was later comparable between patients with reduced and improved EF (Fig. 5A).

Patients with persistent EF had the most favorable prognosis during the follow-up period. However, the differences in overall survival assessed by the likelihood ratio test were not statistically significant (*p* = 0.337096). The analysis showed an increased risk of all-cause death in patients with reduced LVEF (HR 1.896; 0.711 to 5.059) compared to patients with improved EF (HR 0.829; 0.325 to 2.114). At the same time, there was no statistically significant difference between the groups. However, the lack of differences may be due to the small size of the groups.

The analysis showed that metabolomic cluster 4 was associated with the lowest survival, with the majority of deaths occurring in the first year of follow-up (Fig. 5B). The 75th percentile of survival in the Cluster 4 group was 90 days from the start of follow-up (95% CI: 59 to ∞ days). Cluster 3 was characterized by a high mortality rate within 1 year, but the three-year survival rate in this group was the highest. The 75th percentile of survival in the Cluster 3 group was 180 days from the start of follow-up (95% CI: 59 to ∞ days). Cluster 1 and 2 showed a more stable course of the disease and a higher survival rate than cluster 4. For the first cluster, the 75th percentile of survival was 757 days from the start of follow-up (95% CI: 302–1040 days), for cluster 2 it was 912 days from the start of follow-up (95% CI: 151 – ∞ days) (Fig. 5B). However, the differences in overall survival between clusters as assessed by the likelihood ratio test were not statistically significant (*p* = 0.082075). To assess the relationship of clusters with overall survival, the Cox regression method was used.

The all-cause risk ratio for cluster 4 was significantly higher compared to other clusters (HR 2.586; 1.047–6.386) *p* = 0.041. In the presence of metabolomic cluster 4, the risk of mortality increased by 2,57 times. Cluster 3 was also characterized by a high risk of mortality (HR 1.995; CI 0.986–4.036) *p* = 0.04. (Table S10). With cluster 3, the risk of death increased by a factor of 2,119. Age was also a significant factor, increasing the risks by 1,06 times.

The analysis showed a significant increase in the risk of all-cause mortality for clusters 3 and 4. Patients belonging to clusters 3 and 4 have the worst prognosis. Thus, clustering based on metabolomic profiling makes it possible to predict the course of HF.

To determine the prognostic significance of a combination of factors: alternation of LV EF and metabolomic clusters, a model was built that included three variants of the EF trajectory during follow-up, sex, age, and metabolomic clusters. Using the Cox regression method, the assessment of the relationship between metabolomic clusters and EF alternation with overall survival made it possible to construct the following model of proportional risks (Table S11).

**Fig. 6:**
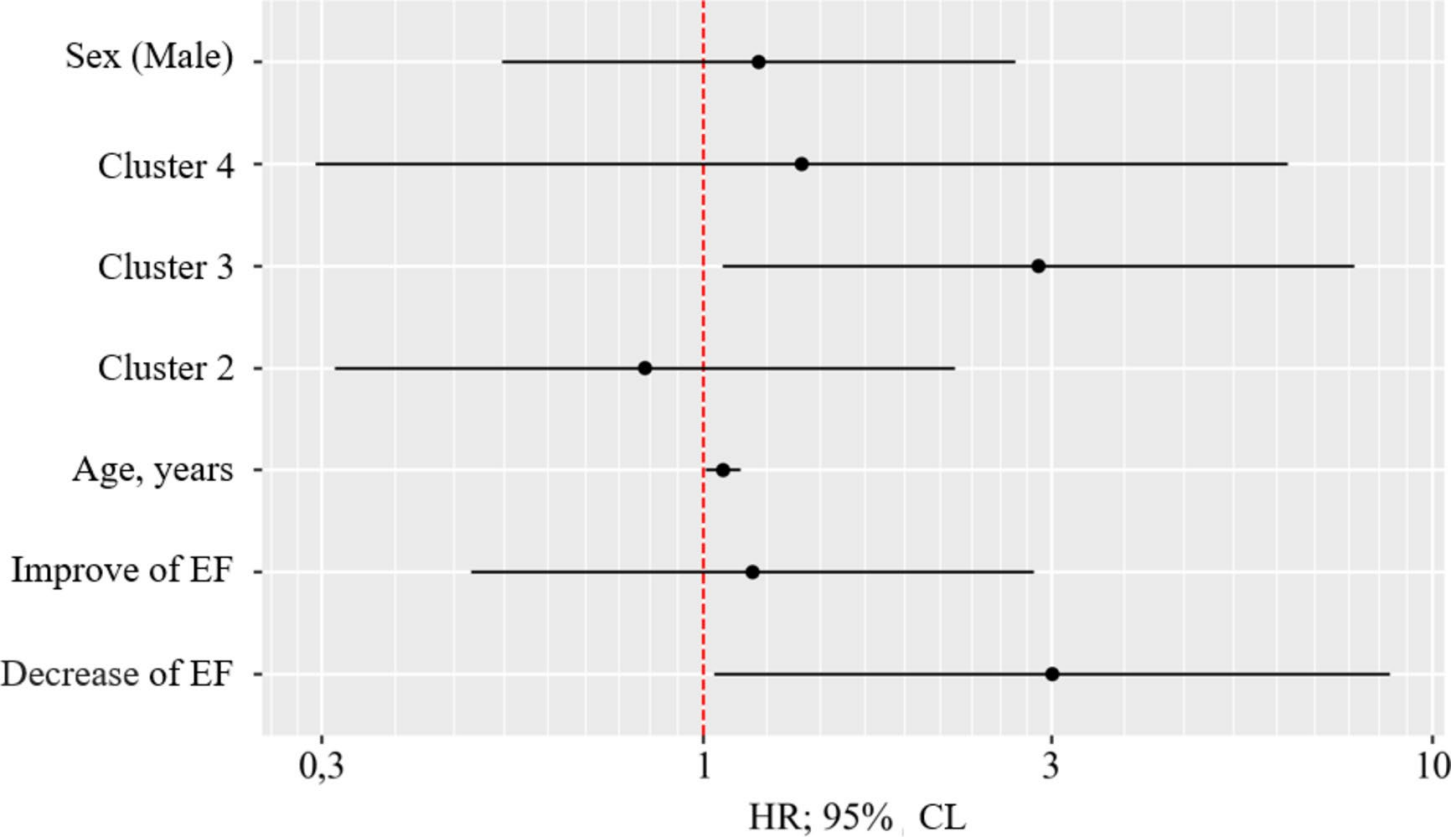
Hazard ratio estimates with 95% CI for the risk factors of all cause death. EF - ejection fraction; male - male sex.

The factors that significantly influenced the prognosis in the model were age, decrease in EF, and the presence of cluster 3. A downward change in the trajectory of EF is associated with a threefold increase in the risk of death from all causes. Cluster 3 was associated with a 2,88-fold increase in all-cause mortality (Fig. 6).

## Discussion

To our knowledge, this is the first study to show a difference of metabolomic profile in the grope of patients with HF, who was reclassified by few principles. ML methods were used as the most promising method for assessing metabolomic data^7^.

### Metabolomic profiling of HF stages

The classification of HF by stages is critically important for screening patients at higher risk of developing HF. Early detection of these patients and preventing disease progression are vital in managing HF (Bozkurt 2023). Studies have shown that pre-HF in the general population often goes unidentified (Bergamasco и др. 2022). This is due to several reasons: the lack of symptoms that prompt patients to seek medical attention and certain limitations related to diagnostic methods themselves. According to the ACC/AHA 2022 guidelines, diagnosing stage B HF requires the assessment of numerous parameters. This approach is highly inconvenient, time-consuming, and impractical for widespread use in clinical practice^8^. Furthermore, several large population-based studies have found that the degree of variation in echocardiographic measurements can reach up to 20% due to subjectivity (McGowan and Cleland 2003). Additionally, natriuretic peptide levels, while useful, are influenced by many factors, and even in the diagnosis of HFpEF, their elevation is not a mandatory criterion. Therefore, from a clinical perspective, diagnosing “pre-HF” poses significant challenges, and the search for markers with high sensitivity and specificity for detecting pre-HF remains a key focus of research. Our results support metabolomic profiling as a promising technique for diagnosing pre-HF with high accuracy, both when compared to stage A and stage C.

Metabolomic profiling across various stages of HF revealed significant differences. Compared to stage A, patients with stage B HF were characterized by: decreased levels of neurotransmitters; increased levels of asymmetric dimethylarginine and symmetric dimethylarginine, associated with atherosclerosis; elevated levels of metabolites from the kynurenine pathway of tryptophan catabolism, linked to the activation of pro-inflammatory cytokines; increased levels of short- and long-chain acylcarnitines (AC); and elevated levels of trimethylamine N-oxide^9,10^. Comparative analyses of stage B and stage C groups showed that symptomatic HF is characterized by a distinct metabolomic profile: a decrease in metabolites involved in the formation of NO (reflected by decreased arginine and its metabolites), which regulates cardiac function through vascular-dependent effects, as well as modulating inflammation and cardiac contractility^11^. Similarly, in our study, patients with stage D HF exhibited disruptions in the glutamate-ornithine-proline cycle and decreased levels of histidine, arginine, and glutamine, indicating a shift in energy balance toward glycolysis^12^.

In summary, our study identified a gradual decline in NO formation, activation of inflammation, elevation of oxidative stress and a shift in energy metabolism characterized by reduced fatty acid metabolism and enhanced anaplerosis and glycolysis during HF progression.

### Metabolomic profiling of HF phenotypes by EF

The EF-based classification method proposed in 2016, which identifies three phenotypes of HF based on LVEF, is essential in clinical practice^13^. However, subsequent years have shown that HFmrEF bears features of both HFpEF and HFrEF^14^. Randomized clinical trials have not provided convincing evidence for the efficacy of specific drug classes in this group^15–18^. As a result, clinical guidelines for managing patients with HFmrEF recommend an approach similar to that for HFrEF, albeit with a lower level of evidence^2,3^. Recently, the relevance of the current classification has been increasingly questioned (Christersson et al. 2024; Seo 2023). At the same time, it remains unclear how patients with HFmrEF should be reclassified and whether such reclassification is pathophysiologically justified.

An important finding of our study is the similarity between the metabolomic profiles of patients with HFmrEF and those with HFrEF, as well as their significant differences from the profiles of patients with HFpEF. This observation underscores the fundamental differences at the cellular and molecular levels between HFpEF and HF with LVEF < 50%. These findings challenge the distinctiveness of the HFmrEF phenotype and provide compelling evidence for reassessing the current EF-based classification. We advocate for delineating two primary phenotypes: HFpEF and HFrEF (EF < 50%). Understanding these mechanisms opens new avenues for developing targeted therapies aimed at correcting metabolic disorders, which could improve the quality of life and prognosis for patients with HF.

Our data align fully with the study by Wynn G. Hunter et al., which compared the metabolomic profiles of patients with HF and EF > 45% to those with systolic dysfunction (EF < 45%). They found that long-chain AC levels were elevated in patients with HFrEF compared to those with HFpEF and significantly differed from the levels in healthy volunteers^19^. In our study, significant differences were also observed in the concentrations of lysine, leucine, and metabolites of the glutamine cycle, with amino acid elevations being particularly notable in the HFpEF group. These findings are consistent with data from other researchers^9,20^. A study by colleagues in China identified significant differences in the metabolomic profiles of patients with HFpEF, HFmrEF, and HFrEF^21^. However, the ML approach used in our research showed low accuracy in distinguishing patients among these three groups.

According to our data, the HFpEF group was characterized by heightened sympathetic-adrenal system activity and exacerbated oxidative stress. It has been established that chronic sympathetic activation leads to ROS-mediated initiation of mitochondrial-dependent cell death cascades^22,23^.

### Metabolomic profiling for clustering HF

Using targeted, quantitative metabolomic profiling, clustering of patients with symptomatic HF was performed, resulting in the identification of four distinct metabolomic clusters. The most significant contributors to cluster separation were AC and metabolites of tryptophan catabolism. Previous metabolomic profiling studies have confirmed the association of predominantly long-chain AC with cardiovascular diseases (CVD) and demonstrated their impact on prognosis^24–27^. This is likely due to the critical role of AC in energy metabolism, particularly in fatty acid (FA) oxidation, which accounts for up to 95% of ATP production in the heart^28^. When the balance is disrupted and FA oxidation slows, incompletely oxidized fatty acids accumulate in mitochondria, leading to lipotoxicity and intracellular acidosis. This further inhibits energy production in cardiomyocytes, exacerbates oxidative stress, and ultimately results in organelle degradation, apoptosis, and the loss of viable myocardium^29–31^. In turn, several studies have shown a relationship between AC and HF and systolic dysfunction^32,33^. Moreover, kynurenine metabolites are independent predictors of adverse prognosis, comparable to NT-proBNP^34^. Tryptophan metabolism plays a key role in regulating hyperinflammation and inducing long-term immune tolerance. These effects are mediated by indoleamine 2,3-dioxygenase (IDO), which alters the local and systemic balance of kynurenine and tryptophan. This balance directly influences metabolic and immune signaling pathways that regulate anti-inflammatory responses in cells with IDO activity, such as antigen-presenting cells and epithelial cells. These cells, in turn, influence neighboring cells (e.g., T-lymphocytes), creating a local (and sometimes systemic) environment characterized by elevated kynurenine and reduced tryptophan levels^35^. A recent comprehensive analysis of tryptophan metabolism in 13 chronic inflammatory diseases found that impaired tryptophan metabolism via the kynurenine pathway is a common feature of chronic inflammatory conditions^36^. Growing evidence links tryptophan catabolism to the development of HF, largely due to the role of inflammation in cardiac remodeling and microvascular dysfunction^36–39^.

In summary, a comparative analysis of metabolite concentrations that significantly influenced cluster separation revealed specific characteristics for each cluster:

- **Cluster 1**: Predominance of oxidative stress.
- **Cluster 2**: Progression of oxidative stress and activation of the sympatho-adrenal system.
- **Cluster 3**: Inflammation.
- **Cluster 4**: Significant impairment of NO synthesis, progression of oxidative stress, and inflammation.

### Predictors of all cause death

The disappointing epidemiological data on mortality rates among patients with HF have driven the search for predictors of unfavorable prognosis. Since EF is a dynamic parameter that can increase in response to therapy or decrease with the development of cardiovascular events, changes in its trajectory may serve as an important prognostic factor^40^. It was shown that HFiEF is associated with a 56% reduction in mortality and a 60% decrease in hospitalization rates compared to patients with HFrEF. Interestingly, patients with HFiEF have a better prognosis than even those with initially preserved EF. Conversely, patients with baseline HFpEF who develop a subsequent decrease in EF have the worst prognosis^41^.

The results of our research align with this trend. The three-year risk of death among all patients with HF was 41.9%. The risk of mortality in the group of patients with a decrease in EF of more than 5%, regardless of baseline levels, was 56%, with early mortality occurring predominantly within the first year of follow-up. Improvement in EF during therapy was observed in 35.5% of patients. Patients with HFiEF had the highest three-year survival rate at 69.3%, though this rate declined sharply thereafter. However, the significant increase in mortality observed in the HFiEF group after 1000 days of follow-up should be interpreted with caution due to the small sample size, which may have influenced the results.

An analysis of survival among patients from different metabolomic clusters revealed that cluster 3 is associated with an increased mortality risk, which is highest within the first year of follow-up. This finding suggests the need for intensified therapy and a potential revision of management strategies for this group. Notably, metabolomic clusters did not differ in terms of EF trajectory. Further studies in larger patient cohorts are required to evaluate the sensitivity and specificity of this approach.

### Clinical implementation of metabolomics

Metabolomic profiling allows for the assessment of the therapeutic effects of various treatment methods and provides insights into the mechanisms of action of medications. Several studies have already demonstrated changes in the metabolomic profile in response to physical exercise (the HF-ACTION study)^42^, as well as during pharmacotherapy and the implantation of medical devices^43^. For example, the use of dapagliflozin over 12 weeks compared to placebo in the DEFINE-HF clinical trial showed an increase in the levels of peripheral metabolites enriched with ketone bodies, as well as short- and medium-chain acylcarnitines, potentially indicating some degree of metabolic reprogramming during SGLT2 inhibitor therapy^44^.

Discoveries from metabolomic studies have enabled the development and proposal of new potential therapeutic approaches. For instance, in patients with heart failure with preserved ejection fraction (HFpEF), the infusion of 3-hydroxybutyrate (a ketone body) increased cardiac output by 2 l/min (40%) with an absolute improvement in left ventricular ejection fraction (8%). The observed effects were accompanied by vasodilation and, consequently, stable systemic and pulmonary arterial pressure^45^. Another study demonstrated the cardioprotective effect of pharmacological activation of branched-chain amino acid (BCAA) oxidation^46^.

Metabolomic phenotyping opens up broad opportunities for the further development of personalized treatment approaches, allowing for the consideration of individual metabolic characteristics of patients to enhance the efficacy of therapy^47^.

### Clinical perspectives

To our knowledge, this is the first study to explore the potential of ML based on metabolomic profiling techniques for analyzing HF across the full spectrum of stages and EF phenotypes, as well as the first to apply clustering to HF using metabolomic profiling. Modern diagnostic methods, combining mass spectrometry with ML, open new avenues for the precise identification of HF subtypes, the discovery of unique phenotypic characteristics, more accurate prediction of disease prognosis and complication risks, and automated analysis of large datasets. These AI-driven supervised ML classification tools offer novel diagnostic options independent of traditional classifications.

Clustering data are particularly valuable as they reveal new HF phenotypes with distinct pathophysiological mechanisms, providing a foundation for developing targeted treatment approaches for these patient subgroups.

Overall, our data are consistent with the findings of other researchers and underscore the importance of changes in EF trajectory as a key factor in predicting adverse clinical outcomes.

### Limitations

The main limitation of this study is the relatively small sample size, which may affect the accuracy of the ML classification model. Repeated echocardiography was not performed for all study participants but only for those who regularly visited the center during the observation period. Consequently, data on changes in ejection fraction over time may be incomplete, limiting the assessment of temporal dynamics.

## Conclusions

The plasma metabolome reflects the primary metabolic changes occurring in the body and serves as a phenotypic imprint. The metabolic similarities observed between HFrEF and HFmrEF phenotypes highlight the limitations of the current EF-based classification and suggest the need for its revision, potentially consolidating patients into two main groups. At the same time, a decrease in LVEF by 5% or more is an important factor associated with an unfavorable prognosis and should be considered in clinical practice. The high diagnostic accuracy for detecting HF stages B and C using ML-based metabolomic profiling supports the effectiveness of this approach for diagnostic purposes.

HF phenotyping through hierarchical clustering based on metabolomic profiles demonstrated high model accuracy. Further studies are needed to clarify the role of metabolomic profiling as a diagnostic tool in HF and to identify patients who may benefit from metabolism-targeted therapies. The findings confirm that metabolomic profiling methods represent a promising alternative for patient stratification and disease classification.

## Electronic supplementary material

Below is the link to the electronic supplementary material.

Supplementary Material 1

## Data Availability

All data generated and analysed during this study are included in this published article and its Supplementary Information files.

## Abbreviations

АC: acylcarnitines
ACEi: angiotensin-converting enzyme inhibitor
ARB: angiotensin receptor blocker
ARNI: angiotensin receptor–neprilysin inhibitor
AT: adenosine triphosphate
AUC ROC: area under the receiver operating characteristics curve
BMI: body mass index
BNP: brain natriuretic peptide
CAD: coronary artery disease
CABG: coronary artery bypass grafting
CCB: calcium channel blocker
СМС: cardiomyocyte
cGMP: cyclic guanosine monophosphate
CVD: cardiovascular diseases
CRP: C-reactive protein
e’: early diastolic velocity of movement of the fibrous ring of the mitral valve
EF: ejection fraction
eGFR: estimated glomerular filtration rate
FA: fatty acids
GDMT: guideline-directed medical therapy
HCA: hierarchal cluster analysis
HDL: high-density lipoprotein
HF: heart failure
HFiEF: heart failure and improved ejection fraction
HFmrEF: heart failure with mildly reduced ejection fraction
HFpEF: heart failure with preserved ejection fraction
HFrEF: heart failure with reduced ejection fraction
IDO: indolеamine 2,3-dioxygenase
LA: left atrium
LAVI: left atrium volume index
LDL: low-density lipoprotein
LC-MS/MS: liquid chromatography mass spectrometry
LV: left ventricular
LVEF: left ventricular ejection fraction
LVMI: left ventricular mass index
MI: myocardial infarction
ML: machine learning
MР: metabolomic profiling
MRA: mineralocorticoid receptor antagonist
NO: nitric oxide
NT-proBNP: N-terminal pro-B-type natriuretic peptide
PCA: principal components analysis
PCI: рercutaneous coronary intervention
QC: quality control
RWT: relative wall thicknes
sGC: soluble guanylate cyclase
SGLT2i: sodium/glucose cotransporter-2 inhibitors
SPAP: systolic pulmonary artery pressure
TR: tricuspid regurgitation
TIA: transient ischemic attack
WBC: white blood counts
