# Supplementary material for: Metabolomic profiling in heart failure as a new tool for diagnosis and phenotyping
Source: Sci Rep. 2025 Apr 7;15:11849. doi: 10.1038/s41598-025-95553-2 (PMC11976976; doi:10.1038/s41598-025-95553-2)
Supplement: Supplementary file 1 — Supplementary Material 1 [file 41598_2025_95553_MOESM1_ESM.docx]

**Supplemental Material**

**Metabolomic Profiling in Heart Failure as A New Tool for Diagnosis and Phenotyping**

**Authors:** Maria V. Kozhevnikova^1*^, PhD, Yuri N. Belenkov^1^, Prof., Ksenia M. Shestakova^2^, PhD, Anton A. Ageev^1^, Pavel A. Markin^2^, PhD, Anastasiia V. Kakotkina^1^, PhD, Ekaterina O. Korobkova^1^, PhD, Natalia E. Moskaleva^2^, PhD, Ivan V. Kuznetsov^1^, Natalia V. Khabarova^1^, PhD, Alexey V. Kukharenko^2^, Svetlana A. Appolonova^2^, PhD.

**Affiliations**: ^1^Hospital Therapy No. 1 Department, Federal State Autonomous Educational Institution of Higher Education I.M. Sechenov First Moscow State Medical University of the Ministry of Health of the Russian Federation (Sechenov University), 119435 Moscow, Russia

^2^Laboratory of Pharmacokinetics and Metabolomic Analysis, Institute of Translational Medicine and Biotechnology, Federal State Autonomous Educational Institution of Higher Education I.M. Sechenov First Moscow State Medical University of the Ministry of Health of the Russian Federation (Sechenov University), 119435 Moscow, Russia

Correspondence to: Maria V. Kozhevnikova, E-mail: kozhevnikova-m@inbox.ru


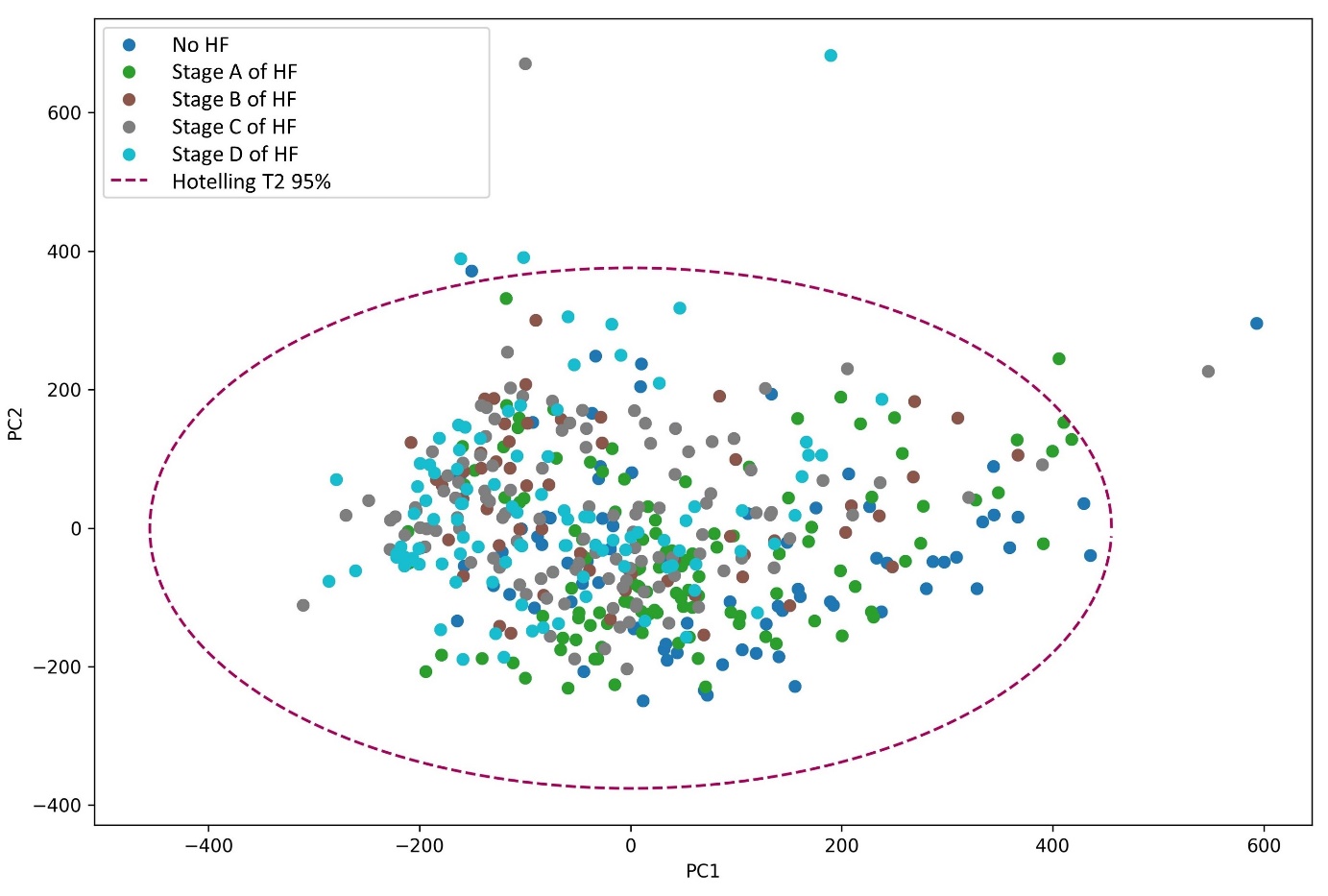


**Figure S1. Principal component analysis for the initial classification of CVD patients.**

CVD - cardiovascular diseases; HF - heart failure.

**Supplemental Tables**

**Table S1. LC-MS\MS parameters.**

| **Name** | **Retention time, min** | **Precursor ion** | **Product ion** | **Frag,V** | **CE,V** | **Internal Standard** |
| --- | --- | --- | --- | --- | --- | --- |
| Quinolinic | 0.35 | 168,1 | 106.1 | 95 | 15 | Quinolinic-*d_3_* |
| Quinolinic-*d_3_* | 0.35 | 171.1 | 109.1 | 95 | 15 |  |
| Betaine | 0.37 | 118.1 | 59.2 | 100 | 25 | Quinolinic-*d_3_* |
| Choline | 0.5 | 104.1 | 60.1 | 100 | 25 | Quinolinic-*d_3_* |
| Dimethylglycine | 0.54 | 104.1 | 58.1 | 95 | 20 | Quinolinic-*d_3_* |
| C0-Carnitine | 0.58 | 162.2 | 103.1 | 85 | 17 | C0-carnitine -*d_9_* |
| C0-carnitine -*d_9_* | 0.58 | 171 | 103 | 85 | 17 |  |
| Creatinine | 0.6 | 114.1 | 44.1 | 90 | 10 | Quinolinic-*d_3_* |
| TMAO | 0.6 | 76 | 58 | 100 | 25 | Quinolinic-*d_3_* |
| Cytidine | 0.98 | 244.1 | 112.1 | 60 | 5 | Quinolinic-*d_3_* |
| C2-carnitine | 1.06 | 204 | 85 | 90 | 22 |  |
| C2-carnitine -*d_3_* | 1.06 | 207 | 85 | 90 | 22 | C2-carnitine -*d_3_* |
| Adenosine | 1.35 | 268.2 | 136 | 100 | 20 | C2-carnitine -*d_3_* |
| Pantothenic | 1.4 | 220.2 | 90 | 100 | 14 | C2-carnitine -*d_3_* |
| Taurine | 1.4 | 261.2 | 126.1 | 100 | 25 | C2-carnitine -*d_3_* |
| Xanturenic acid | 1.5 | 206 | 160 | 95 | 20 | Xanturenic acid-*d_4_* |
| Xanturenic acid-*d_4_* | 1.5 | 210.1 | 164 | 95 | 20 |  |
| C3-carnitine | 1.59 | 218 | 85 | 90 | 23 | C3-carnitine – *d_3_* |
| C3-carnitine – *d_3_* | 1.59 | 221 | 85 | 90 | 23 |  |
| Riboflavin | 1.6 | 377.3 | 243.2 | 100 | 20 | C3-carnitine – *d_3_* |
| Kynurenic acid | 1.6 | 190,1 | 144,1 | 95 | 20 | Kynurenic acid – *d_3_* |
| Kynurenic acid – *d_3_* | 1.6 | 195.1 | 149.1 | 95 | 20 |  |
| HIAA | 1.62 | 192.1 | 146.1 | 100 | 20 | HIAA*-d_5_* |
| HIAA*-d_5_* | 1.62 | 197.1 | 150.1 | 100 | 20 |  |
| C5:1-carnitine | 1.65 | 244 | 85 | 110 | 27 | C5- carnitine- *d_9_* |
| C4-carnitine | 1.69 | 232 | 85 | 90 | 26 | C4-carnitine-*d_3_* |
| C4-carnitine-*d_3_* | 1.69 | 235 | 85 | 90 | 26 |  |
| Histidine | 1.7 | 291.2 | 110 | 100 | 25 | Alanine-*d_4_* |
| Hydroxyproline | 1.7 | 267.1 | 68 | 100 | 25 | Proline- *d_7_* |
| Carnosine | 1.7 | 362 | 110 | 100 | 25 | Alanine-*d_4_* |
| C5-DC-carnitine | 1.7 | 276 | 85 | 110 | 27 | C5- carnitine- *d_9_* |
| Asparagine | 1.71 | 268.2 | 87 | 100 | 25 | Aspartic-*d_3_* |
| Glutamic | 1.72 | 282.2 | 130 | 100 | 25 | Glutamine-*d_5_* |
| Methylhistidine | 1.74 | 305.1 | 212.2 | 100 | 25 | Alanine-*d_4_* |
| Histamine | 1.75 | 247.1 | 154 | 95 | 20 | Alanine-*d_4_* |
| Citrulline | 1.76 | 311.2 | 113.1 | 16 | 16 | Citrulline- *d_2_* |
| Citrulline- *d_2_* | 1.76 | 313.2 | 115.1 | 16 | 16 |  |
| C5-carnitine | 1.76 | 246 | 85 | 110 | 27 | C5- carnitine- *d_9_* |
| C5- carnitine- *d_9_* | 1.76 | 255 | 85 | 110 | 27 |  |
| Arginine | 1.77 | 310.2 | 217.2 | 100 | 25 | Arginine- *d_7_* |
| Arginine- *d_7_* | 1.77 | 317.2 | 224.2 | 100 | 25 |  |
| Serine | 1.78 | 241.2 | 60 | 95 | 20 | Arginine- *d_7_* |
| Methionine-sulfoxide | 1.8 | 301.2 | 88.1 | 100 | 25 | Methionine- *d_3_* |
| Homoarginine | 1.8 | 324.2 | 84.1 | 95 | 20 | Arginine- *d_7_* |
| SDMA | 1.8 | 338.2 | 172 | 100 | 25 | Arginine- *d_7_* |
| ADMA | 1.81 | 338.2 | 46 | 100 | 25 | Arginine- *d_7_* |
| Glycine | 1.82 | 211.2 | 76 | 95 | 10 | Glycine-*13C_2_15N* |
| Glycine-*13C_2_15N* | 1.82 | 214.2 | 79 | 95 | 10 |  |
| Glutamic | 1.84 | 283.2 | 130 | 100 | 25 | Glutamic-*d_5_* |
| Glutamine-*d_5_* | 1.84 | 288.2 | 135 | 100 | 25 |  |
| Aspartic | 1.85 | 269.2 | 116.1 | 100 | 25 | Aspartic-*d_3_* |
| Aspartic-*d_3_* | 1.26 | 137 | 119.1 | 20 | 13 |  |
| Indole-3-lactic | 1.86 | 206.1 | 118.1 | 95 | 22 | Indole-3-lactic-*d_5_* |
| Indole-3-lactic-*d_5_* | 1.86 | 211.2 | 122 | 95 | 22 |  |
| Threonine | 1.9 | 74 | 56 | 20 | 13 | Phenylalanine-*d_5_* |
| Melatonin | 1.9 | 233.2 | 174 | 100 | 15 | Melatonin*-d_4_* |
| Melatonin*-d_4_* | 1.9 | 237.2 | 178 | 100 | 15 |  |
| C6-carnitine | 1.9 | 260 | 85 | 120 | 27 | C6 -carnitine- *d_3_* |
| C6 -carnitine- *d_3_* | 1.9 | 263 | 85 | 120 | 27 |  |
| Proline | 1.95 | 251.2 | 70.3 | 95 | 20 | Proline- *d_7_* |
| Proline- *d_7_* | 1.95 | 258.2 | 77.3 | 95 | 20 |  |
| Indole-3-carboxaldehyde | 1.95 | 146,1 | 118,1 | 95 | 15 | Indole-3-carboxaldehyde-13C_8_ |
| Indole-3-carboxaldehyde-13C_8_ | 1.95 | 154.1 | 126.1 | 95 | 30 |  |
| Alanine | 1.98 | 225.2 | 44.2 | 95 | 20 | Alanine-*d_4_* |
| Alanine-*d_4_* | 1.98 | 229.2 | 48 | 95 | 20 |  |
| Indol-acetic | 2 | 176.1 | 130.1 | 95 | 20 | Indol-acetic- *d_4_* |
| Indol-acetic- *d_4_* | 2 | 180.1 | 133.1 | 95 | 20 |  |
| C8:1 -carnitine | 2.04 | 286 | 85 | 120 | 27 | C8- carnitine-*d_3_* |
| Tyrosine | 2.1 | 317.2 | 165 | 100 | 23 | Tyrosine- *d_4_* |
| Tyrosine- *d_4_* | 2.1 | 321.1 | 140 | 100 | 23 |  |
| Tryptamine | 2.1 | 295.1 | 144,1 | 95 | 20 | Tyrosine- *d_4_* |
| Cortisol | 2.1 | 363 | 121 | 120 | 20 | Melatonin*-d_4_* |
| 3-indolepropionic | 2.19 | 190.1 | 130.1 | 95 | 20 | 3-indolepropionic-*d_2_* |
| 3-indolepropionic-*d_2_* | 2.19 | 192.1 | 130.1 | 95 | 20 |  |
| C8-carnitine | 2.2 | 288 | 85 | 120 | 27 | C8- carnitine-*d_3_* |
| C8- carnitine-*d_3_* | 2.2 | 291 | 85 | 120 | 27 |  |
| Serotonin | 2.24 | 312.3 | 160.2 | 100 | 25 | Serotonin-*d_4_* |
| Serotonin-*d_4_* | 2.24 | 316.2 | 164.2 | 100 | 25 |  |
| C10:2 -carnitine | 2.25 | 312 | 85 | 130 | 27 | C10-carnitine- *d_3_* |
| Valine | 2.3 | 253.2 | 72.1 | 100 | 20 | Valine- *d_8_* |
| Valine- *d_8_* | 2.3 | 261.2 | 80.1 | 100 | 20 |  |
| Methionine | 2.3 | 285.1 | 104.2 | 100 | 25 | Methionine- *d_3_* |
| Methionine- *d_3_* | 2.3 | 288.1 | 107.2 | 100 | 25 |  |
| Indol-3-butyric | 2.34 | 204.2 | 144.2 | 100 | 25 | Indol-3-butyric- *d_4_* |
| Indol-3-butyric- *d_4_* | 2.34 | 208.1 | 132 | 100 | 25 |  |
| C10:1 -carnitine | 2.35 | 314 | 85 | 130 | 27 | C10-carnitine- *d_3_* |
| Anthranilic | 2.37 | 273.2 | 120.1 | 95 | 10 | Valine- *d_8_* |
| Ornitine | 2.41 | 403.2 | 310.2 | 100 | 15 | Ornitine- *d_6_* |
| Ornitine- *d_6_* | 2.41 | 409.2 | 316.2 | 100 | 15 |  |
| 2-hydroxytryptophan | 2.44 | 356.2 | 175.1 | 90 | 20 | Tryptophan-*d_5_* |
| Kynurenine | 2.48 | 344.2 | 146 | 100 | 25 | Kynurenine- *d_4_* |
| Kynurenine- *d_4_* | 2.48 | 348.2 | 150.2 | 100 | 25 |  |
| Lysine | 2.49 | 417.2 | 324.2 | 100 | 20 | Lysine-*13C15N* |
| Lysine-*13C15N* | 2.49 | 419.2 | 326.2 | 100 | 20 |  |
| Tryptophan | 2.5 | 340.2 | 188.2 | 100 | 20 | Tryptophan-*d_5_* |
| Tryptophan-*d_5_* | 2.5 | 345.2 | 193.2 | 100 | 20 |  |
| Leu+Isoleucine | 2.51 | 267.3 | 86 | 100 | 25 | Leucine- *d_3_* |
| Leucine- *d_3_* | 2.51 | 270.3 | 89 | 100 | 25 |  |
| C10 -carnitine | 2.52 | 316 | 85 | 130 | 27 | C10-carnitine- *d_3_* |
| C10-carnitine- *d_3_* | 2.52 | 319 | 85 | 130 | 27 |  |
| Phenylalanine | 2.6 | 301.2 | 120.1 | 100 | 25 | Phenylalanine-*d_5_* |
| Phenylalanine-*d_5_* | 2.6 | 306.2 | 125 | 100 | 25 |  |
| C12:1-carnitine | 2.7 | 342 | 85 | 130 | 27 | C12-carnitine- *d_3_* |
| C14-OH-carnitine | 2.82 | 388 | 85 | 130 | 31 | C14-carnitine- *d_3_* |
| C12-carnitine | 2.83 | 344 | 85 | 130 | 27 | C12-carnitine- *d_3_* |
| C12-carnitine- *d_3_* | 2.83 | 347 | 85 | 130 | 27 |  |
| C14:2-carnitine | 2.9 | 368 | 85 | 130 | 31 | C14-carnitine- *d_3_* |
| C14:1-carnitine | 3 | 400 | 85 | 130 | 31 | C14-carnitine- *d_3_* |
| C14-carnitine | 3.1 | 372 | 85 | 130 | 31 | C14-carnitine- *d_3_* |
| C14-carnitine- *d_3_* | 3.1 | 375 | 85 | 130 | 31 |  |
| C18:2-carnitine | 3.2 | 424 | 85 | 130 | 33 | C18-carnitine- *d_4_* |
| C16:1-carnitine | 3.21 | 414 | 85 | 130 | 33 | C16-carnitine- *d_3_* |
| C16-carnitine | 3.4 | 400 | 85 | 130 | 33 | C16-carnitine- *d_3_* |
| C16-carnitine- *d_3_* | 3.4 | 403 | 85 | 130 | 33 |  |
| C16-OH-carnitine | 3.4 | 416 | 85 | 130 | 33 | C16-carnitine- *d_3_* |
| C18:1-OH -carnitine | 3.4 | 442 | 85 | 130 | 33 | C18-carnitine- *d_4_* |
| C16:1-OH-carnitine | 3.5 | 414.3 | 85 | 130 | 33 | C16-carnitine- *d_3_* |
| C18-OH-carnitine | 3.5 | 444 | 85 | 130 | 33 | C18-carnitine- *d_4_* |
| C18-carnitine | 3.6 | 428 | 85 | 130 | 33 | C18-carnitine- *d_4_* |
| C18:1-carnitine | 3.6 | 426 | 85 | 130 | 33 | C18-carnitine- *d_4_* |
| C18-carnitine- *d_4_* | 3.6 | 431 | 85 | 130 | 33 |  |
| Note: ADMA – asymmetrical dimethylarginine, C0 - carnitine, C2 - acetylcarnitine, C3 - propionylcarnitine, C4 - butyrylcarnitine, C5:1 - tiglylcarnitine, C5-DC - glutarylcarnitine, C5-OH – 3-hydroxyisovalerylcarnitine, C6 - hexanoylcarnitine, C6-DC – adipoylcarnitine, C8 - octanoylcarnitine, C8:1 - octenoylcarnitine, C10 - decanoylcarnitine, C10:1 - decanoylcarnitine, C10:2 - decadienonoylcarnitine, C12 - dodecanoylcarnitine, C12:1 - dodecenoylcarnitine, C14 – tetradecanoylcarnitine, C14-1 – tetradecenoylcarnitine, C14:2 - tetradedecadienoylcarnitine, C14-OH - hydroxytetradecanoylcarnitine, C16 - hexadecanoylcarnitine, C16-OH - 3-hydroxypalmitoleylcarnitine, C16:1 - hexadecenoylcarnitine, C16-1-OH – 3-hydroxypalmitoleylcarnitine, C18 - stearoylcarnitine, C18-OH - 3-hydroxyoctaducenoylcarnitine, C18-1 - octadecenoylcarnitine, C18-1-OH – 3-hydroxyctadecenoylcarnitine, C18:2 - linoleylcarnitine, LC-MS\MS - liquid chromatography mass spectrometry, SDMA – symmetrical dimethylargine, TMAO – trimethylamine oxide. | | | | | | |

**Table S2. Tuned hyperparameters of the classification model.**

| **Hyperparameters of the model** | | | | |
| --- | --- | --- | --- | --- |
| Maximum depth | Maximum features | Min_samples leaf | N estimators | Random state |
| 30 | 40 | 1 | 50 | 42 |

**Table S3. Quality control metrics of the classification model.**

| **Quality metrics of the model** | | | | |
| --- | --- | --- | --- | --- |
| recall | 0.65 | | | |
| AUC | 0.89 | | | |
| Accuracy | 0.65 | | | |
| F1-score | 0.65 | | | |
| **Confusion matrix** | | | | |
|  | Stage A | Stage B | Stage C | Stage D |
| Stage A | 16 | 3 | 5 | 0 |
| Stage B | 4 | 5 | 1 | 1 |
| Stage C | 0 | 0 | 18 | 6 |
| Stage D | 0 | 2 | 6 | 12 |

**Table S4. The most significant metabolites involved in the formation of a Stage A HF and Stage B HF.**

| **Metabolites** | **Coefficient** |
| --- | --- |
| Glutamate | 0,095110 |
| Riboflavin | 0,047560 |
| Norepinephrine | 0,046793 |
| Creatinine | 0,046047 |
| Histamine | 0,034574 |
| Kynurenin | 0,034497 |
| Glycine | 0,032025 |
| Threonine | 0,031287 |
| Taurine | 0,029045 |
| C6-DC | 0,028837 |
| Indole-3-lactic acid | 0,028125 |
| Melatonin | 0,027824 |
| Methionine | 0,026730 |
| C4 | 0,024206 |
| Anthranilic acid | 0,023562 |
| C18-OH | 0,023442 |
| Betaine | 0,022095 |
| 5-HTP | 0,018746 |
| Glutamine | 0,016635 |
| C12 | 0,016598 |
| Asparagine | 0,016531 |
| Tryptophan | 0,015343 |
| C12-1 | 0,014085 |
| C16-OH | 0,013590 |
| Dopamine | 0,013142 |
| Adenosine | 0,012323 |
| Citrulline | 0,012302 |
| C14-OH | 0,0122 |
| C14 | 0,011750 |
| Indole-3-carboxaldehyde | 0,011730 |
| C0 | 0,011086 |
| Proline | 0,010919 |
| Note: C0-L is carnitine, C4 is butyrylcarnitine, C6-DC is adipoylcarnitine, C12 is dodecanoylcarnitine, C12-1 is dodecanoylcarnitine, C14 is tetradecadienoylcarnitine, C14-OH is 3-hydroxyhexadecadienoylcarnitine, C16-OH is 3-hydroxyhexadecanoylcarnitine, C18-OH is 3-hydroxyoctaducenoylcarnitine. | |

**Table S5. The most significant metabolites involved in the formation of Stage B HF and Stage C HF.**

| **Metabolites** | **Coefficient** |
| --- | --- |
| C16-1-OH | 0,095401 |
| C18-1-OH | 0,064302 |
| Norepinephrine | 0,061225 |
| 5-methoxytryptamin | 0,053749 |
| Riboflavin | 0,042611 |
| 3-OH-Anthranilic Acid | 0,041393 |
| Melatonin | 0,035239 |
| Serotonin | 0,033065 |
| C14 | 0,032754 |
| C6-DC | 0,028277 |
| 5- Hydroxytryptophan | 0,026701 |
| Dopamine | 0,023301 |
| Glutamine | 0,022210 |
| Phenylalanine | 0,018934 |
| C5-OH | 0,017653 |
| C16-OH | 0,014978 |
| Goarginine | 0,014302 |
| Histidine | 0,014256 |
| Carnosine | 0,014090 |
| Tryptamine | 0,013751 |
| Glycine | 0,013155 |
| Aspartic acid | 0,013062 |
| Creatinine | 0,012976 |
| C18-OH | 0,012808 |
| Kynurenin | 0,012267 |
| C18-1 | 0,011819 |
| Uridin | 0,011703 |
| C14-1 | 0,01136 |
| Serine | 0,010591 |
| Indole-3-carboxaldehyde | 0,010547 |
| Indole-3-propionic acid | 0,01021 |
| Note: C6-DC – adipoylcarnitine, C14 – tetradecanoylcarnitine, C14-1 – tetradecenoylcarnitine, C16-1-OH – 3-hydroxypalmitoleylcarnitine, C18-1 octadecenoylcarnitine, C18-1-OH – 3-hydroxyctadecenoylcarnitine. | |

**Table S6. Significant metabolites of the model when comparing patients with Stage C HF and Stage D HF.**

| **Metabolites** | **Coefficient** |
| --- | --- |
| C18-1 | 0,040640 |
| TMAO | 0,036346 |
| C18-2 | 0,027838 |
| 3-OH-Anthranilic Acid | 0,025888 |
| 5- Hydroxytryptophan | 0,025507 |
| Goarginine | 0,024108 |
| HIAA | 0,023713 |
| Methylhistidine | 0,021181 |
| Kynurenin | 0,020920 |
| Histidine | 0,020240 |
| C16 | 0,020102 |
| Histamine | 0,018047 |
| Alanine | 0,017916 |
| Adrenaline | 0,017626 |
| C8 | 0,017571 |
| Tryptamine | 0,017421 |
| Kynurenic acid | 0,016724 |
| Lysine | 0,016625 |
| Glycine | 0,016401 |
| Hydroxyproline | 0,015635 |
| Arginine | 0,015159 |
| Taurine | 0,014934 |
| 5-methoxytryptamin | 0,014639 |
| Cortisol | 0,014266 |
| Choline | 0,014196 |
| Indole-3-acetic acid | 0,014037 |
| Pantothenic acid | 0,013706 |
| C14-2 | 0,013417 |
| C5-DC | 0,013168 |
| Aspartic acid | 0,012642 |
| C16-1-OH | 0,012577 |
| C12-1 | 0,012322 |
| Tyrosine | 0,012283 |
| C18-OH | 0,012092 |
| C5-1 | 0,010907 |
| Methionine Sulfoxide | 0,010852 |
| Cytidine | 0,010475 |
| Note: TMAO – trimethylamine oxide, C8 – octanoylcarnitine, C10 – decenoylcarnitine, C5-DC – glutarylcarnitine, C6-DC – adipoylcarnitine, C14 – tetradecadadienoylcarnitine , C16 – hexadecanoylcarnitine, C18-1 octadecenoylcarnitine, C18-OH – 3-hydroxyoctaducenoylcarnitine, C18-2 – linoleylcarnitine. | |

**Table S7. Key metabolites forming the classification model of HFpEF vs. HF with EF < 50%.**

| **Metabolites** | **Factors** |
| --- | --- |
| Kynurenic acid | 0,086658 |
| Quinolinic acid | 0,071322 |
| Kynurenin | 0,039199 |
| HIAA | 0,035661 |
| Indole-3-propionic acid | 0,027395 |
| Tryptophan | 0,025699 |
| Glutamate/Glutamine | 0,024962 |
| C5-DC | 0,024053 |
| Betaine | 0,022750 |
| Indole-3-lactic acid/Kynurenine | 0,022182 |
| Norepinephrine | 0,021285 |
| C8-1 | 0,021166 |
| Anthranilic acid | 0,020213 |
| Ornithine | 0,018073 |
| Riboflavin | 0,016169 |
| ADMA | 0,016100 |
| TMAO | 0,015907 |
| Leucine | 0,014023 |
| Lysine | 0,013802 |
| C16-1-OH | 0,013656 |
| C10-2 | 0,013240 |
| C8 | 0,013200 |
| Tryptamine | 0,012809 |
| Cytidine | 0,011663 |
| Indole-3-butyric acid | 0,011422 |
| Melatonin | 0,011078 |
| Arginine | 0,010925 |
| C6-DC | 0,0108 |
| C18-1-OH | 0,010714 |
| C18-1 | 0,010206 |
| C18-2 | 0,01006 |
| Note: ADMA – asymmetrical dimethylarginine, SDMA – symmetrical dimethylargine C8 – octanoyl carnitine, C10 – decenoyl carnitine, C5-DC glutarylcarnitine, C6-DC – adipoylcarnitine, C16-OH – 3-hydroxypalmitoleylcarnitine, C18-1 octadecene carnitine, C18-1-OH – 3-hydroxytadecenenoylcarnitine C18-2 linoleyl carnitine. | |

**Table S8. Significance of metabolites of the classification model of clustering by metabolomic profiling.**

| **Metabolites** | **Coefficient** |
| --- | --- |
| 3-OH-Anthranilic Acid | 0,012187 |
| 5- Hydroxytryptophan | 0,108709 |
| 5-methoxytryptamin | 0,012245 |
| Glutamine | 0,017077 |
| Glutamate | 0,015874 |
| Xanthurenoic acid | 0,044266 |
| Methionine Sulfoxide | 0,015424 |
| Norepinephrine | 0,062169 |
| Riboflavin | 0,097329 |
| Serine | 0,010361 |
| Tryptophan | 0,012440 |
| Quinolinic acid | 0,046849 |
| C10 | 0,014466 |
| C12 | 0,021110 |
| C12-1 | 0,011517 |
| C14 | 0,024074 |
| C14-2 | 0,012508 |
| C16 | 0,012894 |
| C16-1 | 0,013381 |
| C18-1 | 0,020430 |
| C5-1 | 0,016631 |
| C5-DC | 0,017531 |
| C5-OH | 0,091142 |
| C6 | 0,018899 |
| C6-DC | 0,068441 |
| C8-1 | 0,016041 |
| Note: C5-DC glutarylcarnitine, C5-OH – 3-hydroxyisovalerylcarnitine, C6 – hexanoylcarnitine, C6-DC – adipoylcarnitine, C8-1 – octenoylcarnitine, C10 – decanoylcarnitine, C12 – dodecanoylcarnitine, C12-1 – dodecenoylcarnitine, C14 – tetradedecadienoylcarnitine, C 14-2 – tetradedecadienoylcarnitine, C16 – hexadecanoylcarnitine, C 16-1 – hexadecenoylcarnitine, C18-1 octadecenoylcarnitine. | |

**Table S9. Comparative analysis of metabolites in clusters.**

| **Metabolites** | **Cluster 1** | **Cluster 2** | **Cluster 3** | **Cluster 4** | **P-value** |
| --- | --- | --- | --- | --- | --- |
|  | **Mean**  **Minimum**  **Maximum** | | | |  |
| 3-OH-Anthranilic Acid | 0,06  -0,04  2,02 | 0,05  -0,03  1,03 | 0,31  -0,04  1,71 | 0,04  -0,04  0,28 | < 0,000001*  p 3 – 1 < 0,0000 p 3 – 2 = 0,0121 p 4 – 3 = 0,0012 |
| 5- Hydroxytryptophan | -1,68  -3,30  3,22 | 4,05  -1,35  10,79 | 1,59  -2,45  7,50 | 0,56  -3,14  6,58 | < 0,000001* p 2 – 1 < 0,0000 p 3 – 1 < 0,0000 p 4 – 1 = 0,0037 p 3 – 2 = 0,007 p 4 – 2 = 0,0013 |
| 5-methoxytryptamin | -0,09  -0,15  0,02 | -0,05  -0,12  0,05 | -0,05  -0,16  0,02 | -0,07  -0,14  0,11 | < 0,000001* p 2 – 1 < 0,0000 p 3 – 1 < 0,0000 |
| Glutamate | 23,38  -50,50  181,80 | -12,50  -60,08  62,60 | -28,87  -73,07  160,83 | 16,90  -68,31  281,13 | < 0,000001* p 2 – 1 = 0,0009 p 3 – 1 < 0,0000 p 3 – 2 = 0,0341 p 4 – 3 = 0,0272 |
| Glutamine | -64,73  -238,27  87,32 | -197,07  -260,79  -102,13 | -184,97  -249,56  -31,72 | -85,98  -262,49  308,88 | < 0,000001* p 2 – 1 < 0,0000 p 3 – 1 < 0,0000 p 4 – 2 = 0,0005 p 4 – 3 = 0,0033 |
| Methionine | -0,83  -16,88  25,10 | -9,13  -19,80  8,28 | -13,11  -25,50  6,18 | 1,92  -16,22  27,93 | < 0,000001* p 1 – 2 < 0,0000 p 1 – 3 < 0,0000 p 2 – 3 = 0,0215 p 2 – 4 = 0,0095 p 3 – 4 = 0,0005 |
| Norepinephrine | -1,45  -2,07  0,60 | -0,74  -1,46  -0,06 | -1,80  -2,15  -1,12 | -1,38  -2,10  -0,58 | << 0,000001* p 2 – 1 < 0,000 p 3 – 1 < 0,0000 p 3 – 2 < 0,0000 p 4 – 2 = 0,0000 p 4 – 3 = 0,0003 |
| Quinolonic acid | -0,02  -0,18  0,97 | -0,05  -0,15  0,48 | 0,61  -0,07  0,99 | 0,42  -0,07  2,22 | < 0,000001* p 3 – 1 < 0,0000 p 4 – 1 = 0,0003 p 3 – 2 < 0,000 p 4 – 2 = 0,0003 p 4 – 3 = 0,0393 |
| Riboflavin | -0,03  -0,06  0,13 | -0,03  -0,05  -0,01 | 0,02  -0,05  0,14 | 0,00  -0,05  0,06 | < 0,000001* p 3 – 1 < 0,0000 p 4 – 1 = 0,007 p 3 – 2 < 0,0000 p 4 – 2 = 0,0163 p 4 – 3 = 0,0417 |
| Serin | 1,66  -48,59  94,15 | 7,14  -41,99  67,77 | -21,23  -56,62  47,71 | 5,69  -40,82  83,94 | < 0,000001* p 3 – 1 < 0,0000 p 3 – 2 < 0,0000 p 4 – 3 = 0,0001 |
| Tryptophan | 10,13  -10,40  47,59 | 2,59  -10,69  27,65 | 13,43  -4,99  24,70 | 11,94  -10,44  44,15 | < 0,000001* p 2 – 1 = 0,0016 p 3 – 1 = 0,0041 p 3 – 2 < 0,0000 p 4 – 2 = 0,0041 |
| Xanthurenic acid | 0,01  -0,01  0,13 | -0,01  -0,01  0,02 | 0,01  0,00  0,04 | 0,01  -0,01  0,05 | < 0,000001* p 2 – 1 < 0,0000 p 3 – 2 < 0,0000 p 4 – 2 = 0,0000 |
| C10 | -0,02  -0,07  0,14 | -0,02  -0,06  0,08 | 0,00  -0,08  0,10 | 0,09  -0,05  0,29 | < 0,000001* p 4 – 1 < 0,0000 p 4 – 2 < 0,0000 p 4 – 3 = 0,000 |
| C12 | -0,02  -0,06  0,03 | -0,01  -0,05  0,05 | 0,00  -0,05  0,10 | 0,05  -0,03  0,14 | 0,000001* p 3 – 1 = 0,0280 p 4 – 1 = 0,0000 p 4 – 2 = 0,0000 p 4 – 3 = 0,0079 |
| C12-1 | -0,01  -0,03  0,04 | -0,01  -0,02  0,01 | 0,00  -0,03  0,05 | 0,03  -0,02  0,09 | < 0,000001* p 3 – 1 = 0,000 p 4 – 1 < 0,0000 p 3 – 2 = 0,0062 p 4 – 2 = 0,0000 p 4 – 3 = 0,0154 |
| C14-1 | 0,00  -0,04  0,08 | 0,00  -0,03  0,05 | 0,02  -0,04  0,17 | 0,06  -0,02  0,11 | 0,000003* p 4 – 1 = 0,0000 p 3 – 2 = 0,0380 p 4 – 2 = 0,0000 p 4 – 3 = 0,0130 |
| C14-2 | 0,00  -0,02  0,04 | 0,00  -0,01  0,03 | 0,01  -0,02  0,09 | 0,04  -0,01  0,09 | 0,000001* p 3 – 1 = 0,0025 p 4 – 1 = 0,0000 p 4 – 2 = 0,0038 |
| C16 | 0,00  -0,04  0,05 | 0,01  -0,04  0,06 | 0,02  -0,03  0,12 | 0,03  -0,03  0,13 | 0,000734* p 4 –1 = 0,0031 |
| C16-1 | 0,00  -0,01  0,01 | 0,00  -0,01  0,01 | 0,01  -0,01  0,04 | 0,01  0,00  0,03 | < 0,000001* p 2 – 1 = 0,0461 p 3 – 1 = 0,0000 p 4 – 1 = 0,0000 p 4 – 2 = 0,0072 |
| C18-1 | 0,08  -0,06  0,30 | 0,15  -0,02  0,41 | 0,22  -0,05  0,67 | 0,24  -0,01  0,47 | < 0,000001* p 1 – 2 = 0,0000 p 1 – 3 < 0,0000 p 1 – 4 = 0,0009 p 2 – 3 = 0,0306 |
| C5 | 0,01  -0,03  0,11 | 0,01  -0,04  0,11 | 0,00  -0,04  0,11 | 0,05  -0,02  0,15 | 0,000068* p 3 – 1 = 0,0017 p 4 – 2 = 0,0380 p 4 – 3 = 0,0001 |
| C5-1 | 0,00  -0,01  0,01 | 0,00  -0,01  0,02 | 0,00  -0,01  0,01 | 0,01  -0,01  0,04 | 0,000002* p 4 – 1 < 0,0000 p 4 – 2 = 0,0001 p 4 – 3 = 0,0000 |
| C6 | 0,00  -0,04  0,06 | 0,01  -0,03  0,05 | 0,00  -0,03  0,07 | 0,06  -0,04  0,25 | < 0,000001* p 2 – 1 = 0,025 p 4 – 1 < 0,0000 p 4 – 2 = 0,0054 |
| C8 | -0,04  -0,15  0,41 | -0,01  -0,13  0,26 | -0,01  -0,13  0,21 | 0,15  -0,02  0,31 | < 0,000001* p 2 – 1 = 0,0017 p 3 – 1 = 0,0031 p 4 – 1 < 0,0000 p 4 – 2 = 0,0012 p 4 – 3 = 0,0003 |
| Note: C5-DC glutarylcarnitine, C5-OH – 3-hydroxyisovalerylcarnitine, C6 – hexanoylcarnitine, C6-DC – adipoylcarnitine, C8-1 – octenoylcarnitine, C10 – decanoylcarnitine, C12 – dodecanoylcarnitine, C12-1 – dodecenoylcarnitine, C14 – tetradedecadienoylcarnitine, C 14-2 – tetradedecadienoylcarnitine, C16 – hexadecanoylcarnitine, C 16-1 – hexadecenoylcarnitine, C18-1 octadecenoylcarnitine. | | | | | |

**Table S10. Changes in the risk of all cause death depending on the impact of the cluster by metabolomic profile.**

| **Risk factor** | **Unadjusted** | | **Adjusted** | |
| --- | --- | --- | --- | --- |
|  | **HR; 95% CI** | **P-value** | **HR; 95% CI** | **P-value** |
| Age, years | 1,044;  1,009 – 1,080 | 0,013145* | 1,061;  1,022 – 1,102 | 0,001943* |
| Gender: male | 1,318;  0,760 – 2,285 | 0,325425 | 1,680;  0,924 – 3,054 | 0,088926 |
| Metabolomic clusters: Cluster 2 | 0,824;  0,387 – 1,754 | 0,615989 | 0,798;  0,375 – 1,700 | 0,558806 |
| Metabolomic clusters: Cluster 3 | 2,014;  0,994 – 4,079 | 0,051987 | 2,119;  1,036 – 4,332 | 0,039671* |
| Metabolomic clusters: Cluster 4 | 2,586;  1,047 – 6,386 | 0,039393* | 2,571;  1,042 – 6,343 | 0,040347* |

* – the influence of the predictor is statistically significant (p<0.05)

**Table S11. Changes in the risks of death depending on the influence of individual factors.**

| **Risk factor** | **Unadjusted** | | **Adjusted** | |
| --- | --- | --- | --- | --- |
|  | **HR; 95% CI** | **P-value** | **HR; 95% CI** | **P-value** |
| Cluster 2 | 0,976;  0,375 – 2,543 | 0,960164 | 0,832;  0,313 – 2,212 | 0,7117 |
| Cluster 3 | 2,326;  0,882 – 6,135 | 0,088071 | 2,880;  1,062 – 7,810 | 0,0376* |
| Cluster 4 | 1,434;  0,315 – 6,525 | 0,641375 | 1,364;  0,294 – 6,326 | 0,6919 |
| Age, years | 1,028;  0,983 – 1,075 | 0,225647 | 1,064;  1,007 – 1,125 | 0,0282* |
| Gender, male | 1,030;  0,486 – 2,184 | 0,938263 | 1,191;  0,529 – 2,679 | 0,6733 |
| Alternation of EF: EF decreased | 1,726;  0,687 – 4,335 | 0,245158 | 3,008;  1,035 – 8,743 | 0,0430* |
| EF alternation: EF improved | 1,071;  0,454 – 2,527 | 0,875978 | 1,168;  0,481 – 2,835 | 0,7320 |

* – the influence of the predictor is statistically significant (p<0.05), the EF fraction of the emission.

EF - ejection fraction.
